# A Law of Large Numbers for Local Patterns in Schur Measures and a Schur Process

**DOI:** 10.1007/s10959-025-01421-0

**Published:** 2025-06-02

**Authors:** Pierre Lazag

**Affiliations:** 1 Université d’Angers, LAREMA, UMR CNRS 6093, 2 Boulevard Lavoisier, 49045 Angers cedex 01, France; 2 SISSA, via Bonomea 265, 34136 Trieste, Italy

**Keywords:** Random partitions, Random plane partitions, Schur measures, Schur processes, Determinantal point processes

## Abstract

The aim of this note is to prove a law of large numbers for local patterns in discrete point processes. We investigate two different situations: a class of point processes on the one-dimensional lattice including certain Schur measures, and a model of random plane partitions, introduced by Okounkov and Reshetikhin. The results state in both cases that the linear statistic of a function, weighted by the appearance of a fixed pattern in the random configuration and conveniently normalized, converges to the deterministic integral of that function weighted by the expectation with respect to the limit process of the appearance of the pattern.

## Introduction

The Schur measures were introduced by Okounkov in [20] (see also [3] and [13]) and are probability measures on the set of partitions which lead to determinantal point processes on the one dimensional lattice. An example of a Schur measure is the poissonized Plancherel measure, giving rise to the determinantal point process with the discrete Bessel kernel (see, for example, [4, 12] and [1]). In [7], the author proves a weak law of large numbers for local patterns for the Plancherel measure ([7, Lem. 4.4]). Our first main result (Theorem 1.3), is an extension of the latter law of large numbers to a class of Schur measures to which the Plancherel measure belongs.

The second main result of this note (Theorem 1.8) is an analogous weak law of large numbers for local patterns in a model of random plane partitions. The model we consider is an example of a Schur process considered by Okounkov and Reshetikhin in [22]. Schur processes were introduced in the same paper [22] and are two-dimensional and dynamical generalizations of Schur measures which also lead to point processes that reveal to be determinantal (see also [3] and [5]).

Our results extend the global laws of large numbers for models of random partitions obtained in [9] as they also involve local asymptotic behaviors.

Theorems 1.3 and 1.8 fit into a common and general framework we describe in Section 1.1, before moving into details in Sections 1.2 and 1.3.

### A General Framework

#### Description of the Models

We let *E* be the state space. In our examples, we have either $E=\mathbb {Z}$ or $E=\mathbb {Z}\times \frac{1}{2}\mathbb {Z}$, and we simply assume here that *E* is a discrete (i.e., without accumulation point) countable subset of $\mathbb {R}^d$ for a given integer $d\ge 1$. The probability space is the space of configurations $\textrm{Conf}(E):= \{0,1\}^E$, equipped with the usual Borel sigma-algebra generated by the cylinders. We identify the set $\textrm{Conf}(E)$ with the set of all subsets of *E*. A point process on *E* is a probability measure on $\textrm{Conf}(E)$. For a finite subset $m \subset E$, called a *pattern*, we define the random variable

$$\begin{aligned} c_m : \textrm{Conf}(E)&\rightarrow \mathbb {R}\\ X&\mapsto \left\{ \begin{matrix} 1 &  \text {if }m \subset X, \\ 0 &  \text {else.} \end{matrix} \right. \end{aligned}$$

Observe that by the inclusion/exclusion principle, the law of the random variables $c_m$, $m \subset E$ is a pattern, characterizes a given point process. For $x \in \mathbb {R}^d$ and a pattern $m = \{m_1,\dots ,m_l\}$, we write $m+ x := \{\lfloor m_1 + x \rfloor , \dots , \lfloor m_l + x \rfloor \}$, where $\lfloor y \rfloor$ is one of the closest points to $y \in \mathbb {R}^d$ in *E*. We consider a one-parameter family of point processes $(\mathbb {P}_\alpha )_{\alpha >0}$ which admit a local limit behavior: there exists a set $A \subset \mathbb {R}^d$ and a family of point processes $(\mathbb {P}_{\mathcal {S}(u)})_{u \in A}$ such that the following holds

1 $$\begin{aligned} \lim _{\alpha \rightarrow + \infty } \mathbb {E}_{\alpha } [ c_{ m + \alpha u} ] = \mathbb {E}_{\mathcal {S}(u)} [ c_m ], \end{aligned}$$

for all $u \in A$ and all finite $m\subset E$, where $\mathbb {E}_\alpha$ (resp. $\mathbb {E}_{\mathcal {S}(u)}$) denotes the expectation with respect to $\mathbb {P}_\alpha$ (resp. $\mathbb {P}_{\mathcal {S}(u)}$). The convergence (1) can be interpreted as follows: if, for large $\alpha >0$, we zoom locally around the position $\alpha u$ in a configuration distributed according to $\mathbb {P}_\alpha$, the configuration we see behaves as if it were distributed according to $\mathbb {P}_{\mathcal {S}(u)}$.

#### The Result

For a compactly supported continuous function $f : \mathbb {R}^d \rightarrow \mathbb {R}$ and a finite subset $m \subset E$, we form the sum

2 $$\begin{aligned} \Sigma ( f, m, \alpha ) := \frac{1}{\alpha ^d} \sum _{x \in \alpha A \cap E} f \left( \frac{1}{\alpha } x \right) c_{m + x}. \end{aligned}$$

Consider the deterministic integral

3 $$\begin{aligned} I(f,m) := \int _{A} f(u) \mathbb {E}_{\mathcal {S}(u)} [c_m] du. \end{aligned}$$

A general theorem in this context is the following.

##### Theorem

Assume that for all compact $\mathcal {K} \subset A$, we have

4 $$\begin{aligned} \alpha ^d\sup _{u \in \mathcal {K}} \left| \mathbb {E}_\alpha [c_{m + \alpha u} ] - \mathbb {E}_{\mathcal {S}(u)} [c_m] \right| \rightarrow 0 \end{aligned}$$

as $\alpha \rightarrow + \infty$ and that

5 $$\begin{aligned} \textrm{var}_{\alpha } \left( \Sigma ( f, m, \alpha ) \right) := \mathbb {E}_{\alpha } (\Sigma ( f, m, \alpha )- \mathbb {E}_{\alpha } \Sigma ( f, m, \alpha ))^2 \rightarrow 0 \end{aligned}$$

as $\alpha \rightarrow + \infty$. Then for all $\epsilon >0$, we have

6 $$\begin{aligned} \lim _{ \alpha \rightarrow + \infty } \mathbb {P}_\alpha \left( \left| \Sigma (f,m,\alpha ) - I(f,m) \right| > \epsilon \right) = 0. \end{aligned}$$

#### Scketch of the Proof and Some Comments

The proof is as follows. From (1), we have

$$\begin{aligned} \mathbb {E}_\alpha \Sigma ( f, m, \alpha ) = \frac{1}{\alpha ^d} \sum _{x \in \alpha A \cap E } f \left( \frac{1}{\alpha } x \right) \mathbb {E}_{\mathcal {S}(x/\alpha )} [c_m] +o(1) \end{aligned}$$

where the *o*(1) term arises from condition (4), since the function *f* has compact support. One recognizes on the right hand side a Riemann sum for the integral *I*(*f*, *m*). By condition (5), Chebyshev inequality implies the weak law of large numbers (6).

We will prove in great details the same weak law of large numbers for a class of Schur measures (Theorem 1.3), and for a model of random plane partitions (Theorem 1.8). The main points will be to prove that conditions (4) and (5) are satisfied. Our proofs will lie on the fact that both point processes are determinantal point processes, with kernels described by double contour integrals that will reveal to be suitable for asymptotic analysis. We refer to Section 2 for the definition of determinantal point processes.

Both models we consider are models of random partitions. Other general laws of large numbers for discrete models related to random partitions have been established, for example, in [9], but in the case when the pattern *m* is empty. The latter may thus be seen as global laws of large numbers, while Theorems 1.3 and 1.8 involve a mixture of global and local asymptotic behaviors.

### The Law of Large Numbers for Schur Measures

We start in Section 1.2.1 by recalling the definition of the Schur measures on the set of partitions, following [20] (see also [3, 5] and [13] for another approach). We introduce a one-parameter family of symmetric Schur measures and describe their local limit behavior in Section 1.2.2. The law of large numbers is stated in Section 1.2.3.

#### The Schur Measures on the Set of Partitions

Let $\Lambda$ be the algebra over $\mathbb {C}$ of symmetric functions, i.e., of symmetric polynomials with an infinite number of variables (see [16]). A partition $\lambda = (\lambda _1 \ge \lambda _2 \ge \dots )$ is an almost-zero sequence of nonnegative integers, and is identified with a Young diagram. The set of all partitions is denoted by $\mathbb {Y}$. A distinguished basis in $\Lambda$ is formed by the Schur functions $s_\lambda$, indexed by partitions $\lambda \in \mathbb {Y}$. A specialization is an algebra morphism from $\Lambda$ to $\mathbb {C}$. A specialization $\rho$ is said to be Schur–positive if $\rho (s_\lambda ) \ge 0$ for all $\lambda \in \mathbb {Y}$. The Schur measures are defined as follows.

##### [Style1 Style2]Definition 1.1

Let $\rho$, $\rho '$ be Schur–positive specializations. The Schur measure $\mathbb {S}_{\rho ,\rho '}$ is a probability measure on $\mathbb {Y}$ defined by

$$\begin{aligned} \mathbb {S}_{\rho ,\rho '}(\lambda )= C_{\rho ,\rho '} \rho ( s_\lambda ) \rho ' (s_\lambda ), \quad \lambda \in \mathbb {Y}. \end{aligned}$$

The normalizing constant $C_{\rho ,\rho '}$ is given by the Cauchy formula (see [16])

$$\begin{aligned} C_{\rho ,\rho '}^{-1}= &   \sum _{ \lambda \in \mathbb {Y}} \rho (s_\lambda ) \rho ' (s_\lambda ) = \rho \otimes \rho ' \left( \prod _{i,j=1}^{+\infty } \frac{1}{1 - \textrm{x}_i \textrm{y}_j} \right) \\= &   \rho \otimes \rho ' \left( \exp \left( \sum _{k \ge 1} \frac{p_k(\textrm{x}_1,\mathrm {x_2},\dots )p_k(\textrm{y}_1,\mathrm {y_2},\dots )}{k}\right) \right) , \end{aligned}$$

where the $p_k$’s are the Newton power sums (see [16]).

We assumed that the specializations $\rho$ and $\rho '$ are Schur–positive in order to guarantee that the Schur measure $\mathbb {S}_{\rho ,\rho '}$ is a positive measure. Observe now that this assumption might be avoided if one takes two complex conjugated specializations, which leads to the following definition.

##### [Style1 Style2]Definition 1.2

Let $\rho$ be a specialization. The *symmetric Schur measure* $\mathbb {S}_\rho$ associated to $\rho$ is the probability measure on $\mathbb {Y}$ defined by

$$\begin{aligned} \mathbb {S}_{\rho } ( \lambda ) = C_{\rho } |\rho ( s_\lambda ) |^2, \quad \lambda \in \mathbb {Y}. \end{aligned}$$

Again, the normalizing constant is given by the Cauchy formula

$$\begin{aligned} C_\rho ^{-1} = \rho \otimes \overline{\rho } \left( \prod _{i,j=1}^{+ \infty } \frac{1}{1-\textrm{x}_i\textrm{y}_j} \right) . \end{aligned}$$

**Fig. 1:**
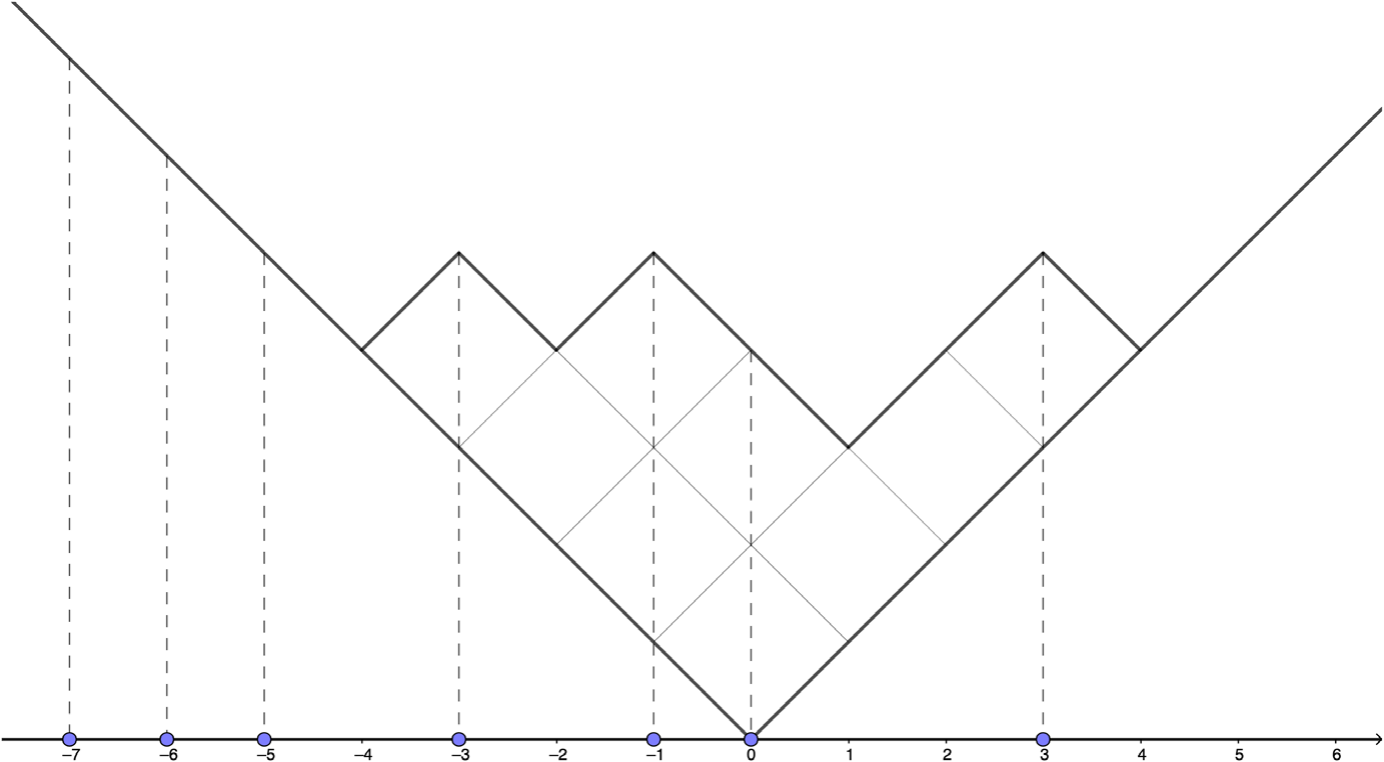
The Young diagramm (4, 2, 2, 1) and its associated configuration

To a partition $\lambda =(\lambda _1 \ge \lambda _2 \ge \dots ) \in \mathbb {Y}$, we associate a configuration $\mathfrak {S}(\lambda ) \in \textrm{Conf}(\mathbb {Z})$ by

$$\begin{aligned} \mathfrak {S}(\lambda ) = \{ \lambda _i - i, \hspace{0.1cm} i=1,2,\dots \}, \end{aligned}$$

see Figure 1 for a picture of the map $\mathfrak {S}$. Okounkov proved in [20] that the image of the Schur measures by the map $\mathfrak {S}$ form determinantal point processes on $\mathbb {Z}$ (see also [3] and [5] for other proofs). We recall this fact in the following Theorem, for the case of symmetric Schur measures.

##### [Style2 Style2]Theorem 1.1

(Okounkov, [20]) The image of the symmetric Schur measure $\mathbb {S}_{\rho }$ by $\mathfrak {S}$, denoted by $\mathbb {P}_\rho$, is a determinantal point process on $\mathbb {Z}$. If the series

$$\begin{aligned} \sum _{k \ge 1} \frac{1}{k} \rho (p_k)z^k \end{aligned}$$

defines a holomorphic function in a neighborhood of the unit circle $\mathbb {T}:= \{ z\in \mathbb {C}, \hspace{0.1cm} |z|=1\}$, then $\mathbb {P}_\rho$ admits the correlation kernel

7 $$\begin{aligned}  &   K_\rho (x,y)\nonumber \\  &   \quad = \frac{1}{(2i \pi )^2} \int _{|z|=1+ \varepsilon } \int _{|w|= 1 - \varepsilon } \exp \left( \sum _{k=1}^{+ \infty } \frac{\rho (p_k)}{k}(z^k- w^{k})+\frac{\overline{\rho (p_k)}}{k}(w^{-k}-z^{-k}) \right) \nonumber \\  &   \quad \frac{1}{z-w}\frac{dzdw}{z^x w^{-y+1}}, \nonumber \\  &   \quad x,y \in \mathbb {Z}, \end{aligned}$$

where $\varepsilon >0$ is sufficiently small.

#### The Limit Processes

Our aim here is to state a local limit behavior for a certain class of Schur measures we now define. Let $\rho$ be a specialization such that the series

$$\begin{aligned} \sum _{k \ge 1} \frac{\rho (p_k)z^k}{k} \end{aligned}$$

defines a holomorphic function in a neighborhood of the unit circle $\mathbb {T}$. Let *G*(*z*) be the function defined by

8 $$\begin{aligned} G(z)= \sum _{k \ge 1} \frac{\rho (p_k)z^k}{k} - \sum _{k \ge 1} \frac{\overline{\rho (p_k)}z^{-k}}{k}. \end{aligned}$$

The function *G* is holomorphic in a neighborhood of $\mathbb {T}$ and we have $G(z) \in i \mathbb {R}$ for all $z \in \mathbb {T}$. We let $\alpha >0$ be a positive parameter, and define the specialization $\rho _\alpha := \alpha \rho$. We denote by $\mathbb {S}_\alpha$ the symmetric Schur measure with specialization $\rho _\alpha$ and let $\mathbb {P}_\alpha$ be its image on $\textrm{Conf}(\mathbb {Z})$ by the map $\mathfrak {S}$.

We can now state a local limit behavior for the point process $\mathbb {P}_\alpha$, as $\alpha \rightarrow + \infty$, Proposition 1.2, analogous to the convergence (1) and (4).

Observe that $zG'(z) \in \mathbb {R}$ when $z \in \mathbb {T}$. Let $u_{\min }$ (resp. $u_{\max }$) be the minimum (resp. maximum) of the function $zG'(z)$, $z \in \mathbb {T}$. For $u \in [u_{\min },u_{\max }]$, the set

$$\begin{aligned} \{z \in \mathbb {T}, \hspace{0.1cm} zG'(z) \ge u \} \end{aligned}$$

consists of a finite number $L=L(u)$ of arcs $[e^{i\phi _k(u)},e^{i\psi _k(u)} ] \subset \mathbb {T}$, $k=1,\dots ,L(u)$. For $u \in [u_{\min },u_{\max }]$, we define the kernel

9 $$\begin{aligned} \mathcal {S}(u)(x,y)&=\sum _{k=1}^{L(u)} \frac{e^{i\phi _k(u)(y-x)}-e^{i\psi _k(u)(y-x)}}{2i\pi (x-y)}, \quad x,y \in \mathbb {Z}, \hspace{0.1cm} x \ne y, \end{aligned}$$

10 $$\begin{aligned} \mathcal {S}(u)(x,x)&= \sum _{k=1}^{L(u)}\frac{\phi _k(u)-\psi _k(u)}{2\pi }, \quad x \in \mathbb {Z}, \end{aligned}$$

and let $\mathbb {P}_{\mathcal {S}(u)}$ be the determinantal point process with correlation kernel $\mathcal {S}(u)$. The local limit behavior (1) together with the estimate (4) for the Schur measures $\mathbb {P}_\alpha$ are provided by the following Proposition. We denote by $\mathbb {E}_\alpha$ the expectation with respect to $\mathbb {P}_\alpha$, and $\mathbb {E}_{\mathcal {S}(u)}$ stands for the expectation with respect to $\mathbb {P}_{\mathcal {S}(u)}$.

##### Proposition 1.2

For all $\delta \in (0,1)$ and all patterns $m\subset \mathbb {Z}$, there exists $C>0$ such that for all $u \in [u_{\min },u_{\max }]$,

11 $$\begin{aligned} \left| \mathbb {E}_\alpha [c_{m + \alpha u} ] - \mathbb {E}_{\mathcal {S}(u)} [c_m ] \right| \le C \exp \left( - \alpha ^\delta \right) \end{aligned}$$

for all sufficiently large $\alpha >0$.

Observe that if we have $L(u)=1$, the kernel $\mathcal {S}(u)$ defined in Equation (9) reads

$$\begin{aligned} \mathcal {S}(u)(x,y) {=} \frac{e^{i\phi (u)(y-x)} {-} e^{i \psi (u) (y-x) }}{2i \pi (x-y)} {=} e^{i\frac{1}{2}(\phi (u) {+} \psi (u))(y-x)} \frac{\sin \left( \frac{\psi (u){-}\phi (u)}{2}(x-y) \right) }{\pi (x-y)}. \end{aligned}$$

Since the factor $e^{i\frac{1}{2}(\phi (u) + \psi (u))(y-x)}$ can be ignored (see Remark 2.1), the point process $\mathbb {P}_\alpha$ converges then to a version of the discrete sine process (see, e.g., [3, 4]).

The class of Schur measures $\mathbb {S}_\alpha$ we consider above are also discussed by Okounkov in the notes [21]. The limit Theorem given by Proposition 1.2 is also stated in the same paper [21] without the error term, and a proof is sketched. We give a complete detailed proof of Proposition 1.2 in Section 1.2.2.

Taking $G(z)=z - z^{-1}$ in Equation (8), one obtains the discrete Bessel point process, the corresponding Schur measure being the poissonized Plancherel measure (see [1, 4, 12] or [3]). In particular, Proposition 1.2 is in that case the convergence of the discrete Bessel point process to the discrete sine process first established in [4].

In Section 3.4, we give an interpretation of the symmetric Schur measures and of the limit kernels $\mathcal {S}(u)$ in terms of shift invariant subspaces of $\ell ^2(\mathbb {Z})$. Namely, we prove that, up to minor transformations, the correlation kernels of symmetric Schur measures are projection kernels onto simply shift invariant subspaces, while the kernels *S*(*u*) are projection kernels onto doubly shift invariant subspaces. Such an interpretation is also given in [8]. Proposition 1.2 provides a connection between both kinds of invariant subspaces.

#### The Law of Large Numbers

As before, we set

$$\begin{aligned} \Sigma (f,m,\alpha )=\frac{1}{\alpha }\sum _{x \in [\alpha u_{\min },\alpha u_{\max }] \cap \mathbb {Z}} f \left( \frac{x}{\alpha } \right) c_{m+x}, \end{aligned}$$

where $f :\mathbb {R}\rightarrow \mathbb {R}$ is a continuous function and $m \subset \mathbb {Z}$ is a pattern. We also define the integral

$$\begin{aligned} I(f,m)= \int _{u_{\min }}^{u_{\max }} f(u) \mathbb {E}_{\mathcal {S}(u)} [c_m] du. \end{aligned}$$

The law of large numbers for Schur measures is the following theorem.

##### [Style2 Style2]Theorem 1.3

For any continuous function $f : \mathbb {R}\rightarrow \mathbb {R}$ and any pattern $m \subset \mathbb {Z}$, we have for all $\epsilon >0$ that

12 $$\begin{aligned} \lim _{\alpha \rightarrow +\infty } \mathbb {P}_{\alpha } \left( \left| \Sigma (f,m,\alpha ) - I(f,m) \right| > \epsilon \right) = 0. \end{aligned}$$

Theorem 1.3 was first established for the discrete Bessel point process in [7, Lem. 4.4], by means of simple estimations of the Bessel functions. The goal of the author in [7] was to prove the Vershik-Kerov entropy conjecture for the Plancherel measure, and it would be intersting to see whether our result could serve to define the entropy of more general Schur measures.

### The Law of Large Numbers for Plane Partitions

We now prepare the statement of the law of large numbers for plane partitions (Theorem 1.8). We present the model in Section 1.3.1 and give the Okounkov-Reshtikhin determinantal formula in Section 1.3.2. The limit processes are presented in Section 1.3.3, and the law of large numbers is stated in Section 1.3.4.

#### Introduction of the Model

A plane partition is a double nonincreasing sequence of nonnegative integers whith a finite number of nonzero elements. More precisely, $\pi =(\pi _{i,j})_{i,j \ge 1} \in \mathbb {Z}_{ \ge 0}^{\mathbb {Z}_{>0} \times \mathbb {Z}_{>0}}$ is a plane partition if and only if

$$\begin{aligned} \pi _{i+1,j}&\le \pi _{i,j} \text { and } \pi _{i,j+1} \le \pi _{i,j} \text { for all } i,j \in \mathbb {Z}_{>0}, \\ |\pi |&:= \sum _{i,j \ge 1} \pi _{i,j} < +\infty . \end{aligned}$$

For $q \in (0,1)$, we consider the geometric probability measure $\tilde{\mathbb {P}}_q$ on the set of all plane partitions given by

$$\begin{aligned} \tilde{\mathbb {P}}_q(\pi ) = M q^{|\pi |}, \end{aligned}$$

where *M* is the normalization constant given by the MacMahon formula ([26], corollary 7.20.3)

$$\begin{aligned} M=\prod _{n=1}^{+\infty }(1-q^n)^n. \end{aligned}$$

**Fig. 2:**
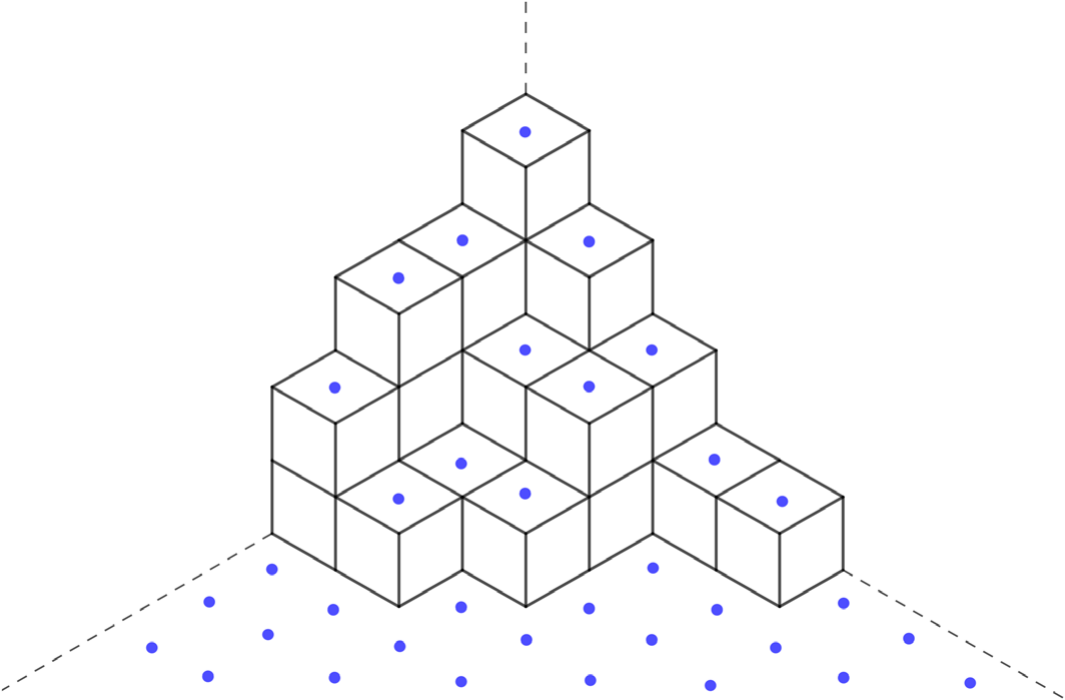
The plane partition $$\begin{pmatrix} 4 &  3 &  2 &  1 &  1 \\ 3 &  2 &  2 &  &  \\ 3 &  1 &  1 &  &  \\ 2 &  1 &  &  &  \end{pmatrix}$$ and its associated configuration

To a plane partition, we associate a subset of $E:=\mathbb {Z}\times \frac{1}{2}\mathbb {Z}$ via the map

$$\begin{aligned} \pi \mapsto \mathfrak {S}_2(\pi ):= \{ (i-j,\pi _{i,j}-(i+j-1)/2), \hspace{0.1cm} i,j \ge 1 \}, \end{aligned}$$

see Figure 2. The first coordinate of a point $(t,h) \in E$ might be interpreted as the time coordinate, and the second as the space coordinate.

The point process we consider, denoted by $\mathbb {P}_q$, is the image of $\tilde{\mathbb {P}}_q$ under $\mathfrak {S}_2$

$$\begin{aligned} \mathbb {P}_q(\mathcal {B}) = \tilde{\mathbb {P}}_q(\mathfrak {S}_2^{-1}(\mathcal {B})), \quad \mathcal {B} \subset \{0,1\}^E \text { is a Borel set}. \end{aligned}$$

#### The Determinantal Formula

Okounkov and Reshetikhin have shown in [22] that $\mathbb {P}_q$ is a determinantal point procces on *E*. We define the kernel $K_q : E \times E \rightarrow \mathbb {R}$ by

13 $$\begin{aligned} K_{q}(t_1,h_1;t_2,h_2)= \frac{1}{(2i\pi )^{2}}\int _{|z|=1 \pm \varepsilon }\int _{|w|=1 \mp \varepsilon } \frac{1}{z-w} \frac{\Phi (t_1,z)}{\Phi (t_2,w)}\frac{dz dw}{z^{h_1+\frac{|t_1|+1}{2}}w^{-h_2-\frac{|t_2|+1}{2}}} \end{aligned}$$

where one picks the plus sign for $t_1 \ge t_2$ and the minus sign otherwise. The function $\Phi$ is defined by

$$\begin{aligned} \Phi (t,z)= {\left\{ \begin{array}{ll} \frac{(q^{1/2}/z;q)_\infty }{(q^{1/2+t}z;q)_\infty } \quad \text {for }t \ge 0, \\ \frac{(q^{1/2-t}/z;q)_\infty }{(q^{1/2}z;q)_\infty } \quad \text {for }t < 0, \end{array}\right. } \end{aligned}$$

where $(x;q)_\infty$ is a *q* version of the Pochhammer symbol

$$\begin{aligned} (x;q)_{\infty }=\prod _{k=0}^{\infty }(1-xq^{k}), \end{aligned}$$

and $\varepsilon$ is a sufficiently small positive number, which allows to avoid the singularities of the ratio

$$\begin{aligned} \frac{\Phi (t_1,z)}{\Phi (t_2,w)}. \end{aligned}$$

Observe that there is no need of defining the square root in formula (13), since, for $(t,h) \in E$, if there exists a plane partition $\pi$ such that $(t,h) \in \mathfrak {S}_2(\pi )$, then we have by construction that

$$\begin{aligned} h+ \frac{|t|+1}{2} \in \mathbb {Z}. \end{aligned}$$

The Okounkov-Reshetikhin determinantal formula is then the following statement

##### [Style2 Style2]Theorem 1.4

(Okounkov-Reshetikhin, [22], 2003) The point process $\mathbb {P}_q$ is a determinantal point process on *E* with correlation kernel $K_q$.

The measure $\tilde{\mathbb {P}}_q$ is a particular case of a Schur process. Schur processes, first introduced in [22], are dynamical generalizations of Schur measures in the sense that they form a Markov process on the set of partitions such that their marginal distributions are Schur measures. For a more elementary treatment of Schur processes, see, e.g., [5, 3] and references therein.

#### The Limit Processes

**Fig. 3:**
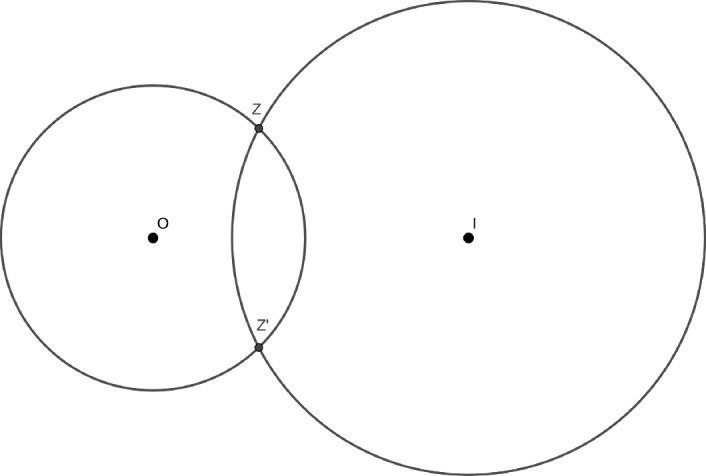
The circles $C(0,e^{-\tau /2})$ and $C(1,e^{-\tau /4-\chi /2})$ and their intersection points $z=z(\tau ,\chi )$ and its complex conjugate $z'=\overline{z(\tau ,\chi )}$

In the same article, Okounkov and Reshetikhin proved a scaling limit theorem for $\mathbb {P}_q$, when $r=-\log (q)$ tends to $0^+$, which we now formulate. Let us define

$$\begin{aligned} A:= \lbrace (t,x) \in \mathbb {R}^{2} , |2\cosh (t/2)-e^{-x}| < 2 \rbrace \subset \mathbb {R}^2, \end{aligned}$$

and for $(\tau ,\chi ) \in A$, let $z(\tau ,\chi )$ be the intersection point of the circles $C(0,e^{-\tau /2})$ and $C(1,e^{-\tau /4-\chi /2})$ with positive imaginary part (see Figure 3). The condition $(\tau ,\chi ) \in A$ guarantees that $z(\tau ,\chi )$ exists and is not real. For $(\tau ,\chi ) \in A$, we define the translation invariant kernel $\mathcal {S}_{z(\tau ,\chi )} : E \rightarrow \mathbb {C}$

14 $$\begin{aligned} \mathcal {S}_{z(\tau ,\chi )}(\Delta t, \Delta h)=\frac{1}{2i\pi }\int _{\overline{z(\tau ,\chi )}}^{z(\tau ,\chi )}(1-w)^{\Delta t}w^{-\Delta h - \frac{\Delta t}{2}} \frac{dw}{w}, \end{aligned}$$

where the integration path crosses (0, 1) for $\Delta t \ge 0$ and $(-\infty ,0)$ for $\Delta t < 0$. For reasons explained below, this kernel will be called the *extended sine kernel*. Then, the following holds

##### [Style2 Style2]Theorem 1.5

(Okounkov-Reshetikhin, [22]) For all $(\tau , \chi ) \in A$ and all patterns $m=\{ (t_1,h_1),...,(t_l,h_l) \} \subset E$, we have

$$\begin{aligned} \lim _{r\rightarrow 0} \mathbb {E}_{e^{-r}} \left[ c_{\frac{1}{r}(\tau ,\chi )+m } \right] = \det \left( \mathcal {S}_{z(\tau ,\chi )}(t_i-t_j,h_i-h_j) \right) _{i,j=1}^l. \end{aligned}$$

For $(\tau , \chi ) \in A$, the kernel $\mathcal {S}_{z(\tau ,\chi )}$ defines a determinantal point process $\mathbb {P}_{(\tau ,\chi )}$ on *E* by

15 $$\begin{aligned} \forall m{=}\{ (m^1_1,m^2_1), ..., (m^1_l,m^2_l) \} \subset E, \quad \mathbb {E}_{(\tau ,\chi )} [c_m] {=} \det \left( \mathcal {S}_{z(\tau ,\chi )}(m_i^1{-}m_j^1,m_i^2{-}m_j^2)\right) _{i,j=1}^l, \end{aligned}$$

where $\mathbb {E}_{(\tau ,\chi )}$ is the expectation with respect to $\mathbb {P}_{(\tau ,\chi )}$. This point process can be seen as a two-dimensional or dynamical version of the usual discrete sine-process on $\mathbb {Z}$ (see, e.g., [4] or [3]). Indeed, setting $\Delta t =0$ in (14) leads to

$$\begin{aligned} \mathcal {S}_{z(\tau ,\chi )}(0,\Delta h) = e^{\frac{\tau \Delta h}{2}} \frac{\sin (\phi \Delta h)}{\pi \Delta h} \end{aligned}$$

where $z(\tau ,\chi )= e^{-\frac{\tau }{2}+i\phi }$. Note that we can ignore the factor $e^{\frac{\tau \Delta h}{2}}$ (see Remark 2.1).

We give the speed of convergence in Theorem 1.5 in the following proposition.

##### Proposition 1.6

For any compact $\mathcal {K} \subset \mathbb {R}^2$, and any pattern $m \subset E$, there exists $C>0$, such that for all $r>0$ sufficiently small and all $(\tau ,\chi ) \in A \cap \mathcal {K}$, one has

16 $$\begin{aligned} |\mathbb {E}_{e^{-r}}[c_{\frac{1}{r}(\tau ,\chi )+m}]-\mathbb {E}_{(\tau ,\chi )}[c_m]| \le Cr. \end{aligned}$$

##### Remark 1.7

We chose here and all along the the rest of the paper that concerns this model of random plane partitions to use the notation of [22]. In order to connect with the notation for the parameters used in the general framework of Section 1.1, we shall set

$$\begin{aligned} \alpha := \frac{1}{r} = -\frac{1}{\log q} \end{aligned}$$

and $$u:=(\tau ,\chi )$$.

The extended sine kernel appears in many other models as the kernel of the bulk scaling limit of two-dimensional statistical mechanics models, for example nonintersecting paths (see, e.g., [11] and references therein). It also has a continuous counter-part arising in the Dyson’s brownian motion model (see, e.g., [14]).

A similar model of random plane partitions has also been considered in [10], and related models of random skew plane partitions and their scaling limits have been studied, for example, in [6, 17, 23] and [18].

#### The Law of Large Numbers

For $r >0$, we define the set $A_{r} \subset E$ by

$$\begin{aligned} A_{r}= r^{-1}A \cap E. \end{aligned}$$

For brevity, we write $\mathbb {P}_r$ (resp. $\mathbb {E}_r$) instead of $\mathbb {P}_{e^{-r}}$ (resp. $\mathbb {E}_{e^{-r}}$). For a continuous compactly supported function $f : \mathbb {R}^2 \rightarrow \mathbb {R}$, and a pattern $m \subset E$, we form the empirical average

17 $$\begin{aligned} \Sigma (f,m,r)=r^2 \sum _{(t,h) \in A_{r} }f(rt,rh)c_{(t,h)+m}, \end{aligned}$$

and we define the deterministic integral

$$\begin{aligned} I(f,m) = \int _{A} f(\tau ,\chi )\mathbb {E}_{(\tau ,\chi )} [ c_m ] d\tau d\chi . \end{aligned}$$

Our theorem establishes that, under $\mathbb {P}_r$, the sum $\Sigma (f,m,r)$ converges to *I*(*f*, *m*).

##### [Style2 Style2]Theorem 1.8

For every continuous compactly supported function $f: \mathbb {R}^2 \rightarrow \mathbb {R}$, every pattern $m \subset E$, one has

18 $$\begin{aligned} \forall \epsilon>0, \quad \lim _{r \rightarrow 0} \mathbb {P}_{r} \left( |\Sigma (f,m,r) - I(f,m) | > \epsilon \right) =0. \end{aligned}$$

##### Remark 1.9

The assumption of compactness of the support of the function *f* is used for the uniformity of constants in estimations of averages and variances. It might be interesting to see whether Theorem 1.8 still holds for a wider class of functions, e.g., Schwartz functions.

### Organization of the Paper

The paper is organized as follows. In Section 2, we recall the definition of a determinantal point process on a discrete space *E*. We show in Proposition 2.2 a way to control covariances for disjoint patterns in a determinantal point process. The proposition will be used in the proofs of Lemma 3.1 and especially in the proof of Lemma 4.1, as it provides a method of symmetrizing the problem posed by the fact that the correlation kernel $K_q$ is not symmetric.

Section 3 is devoted to the proof of Theorem 1.3. We state preliminary results in Section 3.1 concerning the control of the covariances for the symmetric Schur measures, Lemmas 3.1 and 3.2. We prove Theorem 1.3 in Section 3.2, assuming Proposition 1.2 and Lemmas 3.1 and 3.2. We prove Proposition 1.2 in Section 3.3. In Section 3.4, we give an interpretation of Proposition 1.2 in terms of shift invariant subspaces of $l^2(\mathbb {Z})$; this part is rather independent of the rest of the paper. We finally prove the decorrelation Lemmas 3.1 and 3.2 in Section 3.5.

We prove Theorem 1.8 in Section 4, the structure being the same as for the preceding Section 3. We state Lemmas 4.1 and 4.2 concerning the control of the covariances in Section 4.1, before proving Theorem 1.8 in Section 4.2, assuming Proposition 1.6, Lemmas 4.1 and 4.2. We prove Proposition 1.6 in Section 4.3, and the decorrelation lemmas 4.1 an 4.2 in Section 4.4

## Determinantal Point Processes

### Definitions

We recall the following

#### [Style1 Style2]Definition 2.1

A point process $\mathbb {P}$ on *E* is a *determinantal point process* if there exists a kernel

$$\begin{aligned}K : E \times E \rightarrow \mathbb {C}, \end{aligned}$$

called a correlation kernel for $\mathbb {P}$, such that, for any finite $m=\{m_1,\dots ,m_l \} \subset E$, we have

19 $$\begin{aligned} \mathbb {P}( m \subset X) = \mathbb {E}_\mathbb {P}[c_m] = \det \left( K(m_i,m_j) \right) _{i,j=1}^l. \end{aligned}$$

#### Remark 2.1

A correlation kernel of a given determinantal point process is not unique. Indeed, if $\mathbb {P}$ is a determinantal point process with correlation kernel *K*, then for any nonvanishing function $f : E \rightarrow \mathbb {C}$, the kernel

$$\begin{aligned} \tilde{K}(x,y) = \frac{f(x)}{f(y)} K(x,y) \end{aligned}$$

also serves as a correlation kernel for $\mathbb {P}$, since the factors involving the function *f* will disappear from any determinant of the form (19).

A correlation kernel *K* defines an integral operator on $\ell ^2(\mathbb {Z})$, which we denote by the same letter *K*

$$\begin{aligned} K :\ell ^2(\mathbb {Z})&\rightarrow \ell ^2(\mathbb {Z}) \\ f&\mapsto \left( x \mapsto \sum _{y \in E} K(x,y)f(y) \right) . \end{aligned}$$

Conversely, by the Macchi-Soshnikov/Shirai-Takahashi theorem ([15, 24, 25]), any locally trace class hermitian integral operator *K* such that $0 \le K \le I$ gives rise to a determinantal point process. In particular, any orthogonal projection onto a closed subspace $\mathcal {E}$ of $\ell ^2(\mathbb {Z})$ gives rise to a determinantal point process.

### Covariances for Discrete Determinantal Point Processes

We here provide a method for the estimation of covariances for discrete determinantal point processes. In particular, this method will be useful for determinantal point processes with a nonsymmetric correlation kernel.

#### Proposition 2.2

Let $\mathbb {P}$ be a determinantal point process on *E* with correlation kernel *K*. Let $m=\{ m_1,\dots , m_l\}$, $m'=\{m_1',\dots ,m_{l'}' \} \subset E$ be two disjoint patterns. Then, the covariance

20 $$\begin{aligned} \textrm{cov}_\mathbb {P}(c_m,c_m'):= \mathbb {E}_\mathbb {P}[c_m c_{m'} ] - \mathbb {E}_\mathbb {P}[c_m] \mathbb {E}_\mathbb {P}[c_{m'}] \end{aligned}$$

is a sum of $(l+l')! -l!l'!$ terms, each one containing a factor of the form

$$\begin{aligned} K(m_i,m_j')K(m_i',m_j). \end{aligned}$$

#### Proof

Since the patterns *m* and $m'$ are disjoint, we have

$$\begin{aligned} c_mc_{m'} =c_{m \sqcup m'}. \end{aligned}$$

By definition of a determinantal point process, the expectation

$$\begin{aligned} \mathbb {E}_\mathbb {P}[c_mc_{m'}] \end{aligned}$$

is an alternate sum of $(l+l')!$ terms indexed by the permutations of the set $m\sqcup m'$. Among these permutations, let us consider those that leave the sets *m* and $m'$ invariant, i.e., permutations $\sigma$ which can be factored as $\sigma =\tau \tau '$ where $\tau$ (resp. $\tau '$) is a permutation of *m* (resp. $m'$). The alternate sum over all the permutations leaving the sets *m* and $m'$ invariant is nothing but the product of the determinants

$$\begin{aligned} \mathbb {E}_\mathbb {P}[c_m] \mathbb {E}_\mathbb {P}[c_{m'}]. \end{aligned}$$

Thus, the remaining terms in the covariance (20) are all indexed by permutations $\sigma$ leaving neither *m* nor $m'$ invariant (since $\sigma$ is a permutation, if $\sigma$ left *m* invariant, it would also leave $m'$ invariant), i.e., there exist $m_i,m_j \in \{m_1,\dots ,m_l\}$ and $m_i',m_j' \in \{m_1',\dots ,m_{l'}'\}$ such that

$$\begin{aligned} \sigma (m_i)=m_j' \quad \text {and} \quad \sigma (m_{i}')=m_j. \end{aligned}$$

The proof is complete. $\square$

## Proof of Theorem 1.3

### Control of the Covariances

Let $\alpha >0$ and let $\mathbb {P}_\alpha$ be the image by $\mathfrak {S}$ of the symmetric Schur measure $\mathbb {S}_\alpha$ defined in Section 1.2. We start by stating preliminary results on the control of the covariances

$$\begin{aligned} \textrm{cov}_\alpha (c_{m + \alpha u_1},c_{m + \alpha u_2})&= \mathbb {E}_\alpha [c_{m+\alpha u_1} c_{m + \alpha u_2} ] \\&\quad - \mathbb {E}_\alpha [c_{m+\alpha u_1}] \mathbb {E}_\alpha [ c_{m + \alpha u_2} ], \end{aligned}$$

distinguishing the cases when $u_1$, $u_2 \in [u_{\min }, u_{\max }]$ are away from each other or not.

#### Lemma 3.1

Let $m = \{m_1,\dots ,m_l\} \subset \mathbb {Z}$ be a pattern and denote by $\overline{m}$ its supremum norm

$$\begin{aligned} \overline{m} = \max \{|m_i|, \hspace{0.1cm} i=1,\dots ,l\}. \end{aligned}$$

There exists $C>0$ such that for all sufficiently large $\alpha >0$ and for any $u_1,u_2 \in [u_{\min },u_{\max }]$ such that

$$\begin{aligned}|u_1-u_2| > \frac{\overline{m}}{\alpha }, \end{aligned}$$

we have

21 $$\begin{aligned} \left| \textrm{cov}_\alpha (c_{m+ \alpha u_1 }, c_{m + \alpha u_2 }) \right| \le \frac{C}{\alpha |u_1-u_2|}. \end{aligned}$$

#### Lemma 3.2

For any patterns $m,m' \subset \mathbb {Z}$, there exists $C>0$ such that for any $u \in [u_{\min },u_{\max }]$ and any sufficiently large $\alpha >0$ we have

22 $$\begin{aligned} \left| \textrm{cov}_\alpha ( c_{m + \alpha u}, c_{m' + \alpha u}) \right| \le C. \end{aligned}$$

### Proof of Theorem 1.3

For brevety, we write

$$\begin{aligned} A_\alpha := [\alpha u_{\min }, \alpha u_{\max }] \cap \mathbb {Z}. \end{aligned}$$

We start by writing

$$\begin{aligned} \mathbb {E}_\alpha \Sigma (f,m,\alpha ){=} \frac{1}{\alpha } \sum _{x \in A_\alpha } f \left( \frac{x}{\alpha } \right) \mathbb {E}_{\alpha } [ c_{m + x} ] {=} \frac{1}{ \alpha } \sum _{ u \in [ u_{\min } , u_{\max }] \cap \frac{1}{\alpha }\mathbb {Z}} f(u) \mathbb {E}_{\alpha } [c_{m {+} \alpha u} ]. \end{aligned}$$

By Proposition 1.2, and since

$$\begin{aligned} \left| A_\alpha \right| = O \left( \alpha \right) , \end{aligned}$$

we have that, for any $\delta \in (0,1)$, there exists $C>0$ such that

23 $$\begin{aligned} \left| \mathbb {E}_\alpha \Sigma (f,m,\alpha ) - \frac{1}{\alpha } \sum _{ u \in [ u_{\min } , u_{\max }] \cap \frac{1}{\alpha }\mathbb {Z}} f(u) \mathbb {E}_{\mathcal {S}(u)} [c_m] \right| \le C \alpha \exp \left( -\alpha ^{-\delta } \right) . \end{aligned}$$

The sum

$$\begin{aligned} \frac{1}{\alpha } \sum _{ u \in [ u_{\min } , u_{\max }] \cap \frac{1}{\alpha }\mathbb {Z}} f(u) \mathbb {E}_{\mathcal {S}(u)} [c_m] = \frac{1}{\alpha } \sum _{ x \in A_\alpha } f \left( \frac{x}{\alpha } \right) \mathbb {E}_{\mathcal {S}(x/\alpha )} [c_m] \end{aligned}$$

is a Riemann sum for the integral

$$\begin{aligned} I(f,m)= \int _{u_{\min }}^{u_{\max }} f(u) \mathbb {E}_{\mathcal {S}(u)} [c_m] du, \end{aligned}$$

whence we deduce from (23) that

$$\begin{aligned} \lim _{\alpha \rightarrow + \infty } \mathbb {E}_\alpha \Sigma (f,m ,\alpha ) = I(f,m). \end{aligned}$$

It suffices now to prove that the variance

24 $$\begin{aligned} \textrm{var}_\alpha \Sigma (f,m,\alpha )= &   \mathbb {E}_\alpha \left| \Sigma (f,m,\alpha ) - \mathbb {E}_\alpha \Sigma (f,m,\alpha ) \right| ^2 \nonumber \\= &   \frac{1}{\alpha ^2} \sum _{x,y \in A_\alpha } f\left( \frac{x}{\alpha } \right) f \left( \frac{y}{\alpha } \right) \textrm{cov}_{\alpha } (c_{m + x}, c_{m + y}) \end{aligned}$$

goes to 0 as $\alpha \rightarrow + \infty$. We cut the set $A_\alpha ^2$ into two parts by setting

$$\begin{aligned} A_\alpha ^>&= \{(x,y) \in A_\alpha ^2, \hspace{0.1cm} |x-y|> \overline{m} \} , \\ A_\alpha ^\le&= A_\alpha ^2 \setminus A_\alpha ^>, \end{aligned}$$

and decompose the variance (24) into two sums

25 $$\begin{aligned} \textrm{var}_\alpha \Sigma (f,m,\alpha )= &   \frac{1}{\alpha ^2} \left( \sum _{(x,y) \in A_\alpha ^>} f\left( \frac{x}{\alpha } \right) f \left( \frac{y}{\alpha } \right) \textrm{cov}_{\alpha } (c_{m + x}, c_{m + y}) \right. \nonumber \\  &   + \left. \sum _{ (x,y) \in A_\alpha ^\le } f\left( \frac{x}{\alpha } \right) f \left( \frac{y}{\alpha } \right) \textrm{cov}_{\alpha } (c_{m + x}, c_{m + y}) \right) . \end{aligned}$$

By construction, for all $(x,y) \in A_\alpha ^>$, there exists $u_1,u_2 \in [u_{\min },u_{\max }] \cap \frac{1}{\alpha }\mathbb {Z}$ satisfying

$$\begin{aligned} |u_1-u_2| > \frac{\overline{m}}{\alpha } \end{aligned}$$

and such that

$$\begin{aligned} x=\alpha u_1, \quad y = \alpha u_2. \end{aligned}$$

By Lemma 3.1, there exists $C >0$ such that for all $(x,y) \in A_\alpha ^>$, we have

26 $$\begin{aligned} |\textrm{cov}_{\alpha }(c_{m+x},c_{m+ y })| \le \frac{C}{|x-y|}. \end{aligned}$$

Since

$$\begin{aligned}|A_\alpha ^>| =O \left( \alpha ^2\right) , \end{aligned}$$

and since the function *f* is bounded on $[u_{\min },u_{\max }]$, we deduce from (26) that

27 $$\begin{aligned} \sum _{(x,y) \in A_\alpha ^>} \left| f\left( \frac{x}{\alpha } \right) f \left( \frac{y}{\alpha } \right) \textrm{cov}_{\alpha } (c_{m + x}, c_{m + y}) \right| \le C' \sum _{(x,y) \in A_\alpha ^>} \frac{1}{|x-y|} = O \left( \alpha \log (\alpha ) \right) . \end{aligned}$$

Now, for any $(x,y) \in A_\alpha ^\le$, there exist $u \in [u_{\min }, u_{\max }]$ and patterns $m', m'' \subset \mathbb {Z}$ such that

$$\begin{aligned} x+m = \alpha u + m', \quad y+ m = \alpha u + m''. \end{aligned}$$

Observe that there is only a finite number of possibilities for the patterns $m', m''$ as (*x*, *y*) ranges over $A_\alpha ^\le$. Thus by Lemma 3.2, there exists $C>0$ such that for all $(x,y) \in A_\alpha ^\le$, we have

28 $$\begin{aligned} \left| \textrm{cov}_\alpha (c_{m+x,m+y}) \right| \le C. \end{aligned}$$

We obtain from (28) that there exists $C'>0$ such that

29 $$\begin{aligned} \sum _{(x,y) \in A_\alpha ^\le } \left| f\left( \frac{x}{\alpha } \right) f \left( \frac{y}{\alpha } \right) \textrm{cov}_{\alpha } (c_{m + x}, c_{m + y}) \right| \le C'\alpha . \end{aligned}$$

Inserting inequalities (27) and (29) into (25), we obtain

$$\begin{aligned} \textrm{var}_\alpha \Sigma (f,m,\alpha ) = O \left( \frac{\log \alpha }{\alpha } \right) \end{aligned}$$

and the proof is complete.

### Proof of Proposition 1.2

For $u \in [u_{\min },u_{\max }]$, we define the function

$$\begin{aligned} S_u(z) = G(z) - u\log (z), \end{aligned}$$

for *z* in a neighborhood of the unit circle $\mathbb {T}$, except at the intersection with the semi-axis $(-\infty ,0]$, so that the logarithm is well defined. Let $K_\alpha$ be the correlation kernel of the point process $\mathbb {P}_\alpha$. We will prove that

30 $$\begin{aligned} \lim _{ \alpha \rightarrow + \infty } K_\alpha ( \alpha u + x , \alpha u + y) = \mathcal {S}(u)(x,y), \end{aligned}$$

with a remaining term of order at most $\exp \left( -\alpha ^\delta \right)$, $\delta \in (0,1)$. By Theorem 1.1, we have

31 $$\begin{aligned} K_\alpha (x +\alpha u, y+ \alpha u) {=} \frac{1}{(2i \pi )^2} \int _{|z|=1 + \varepsilon } \int _{|w| {=} 1 - \varepsilon } \frac{\exp \left( \alpha \left( S_u(z) - S_u(w) \right) \right) }{z-w} \frac{dzdw}{z^{x}w^{-y+1}}. \end{aligned}$$

We want to deform the contours of integration in order to have $\Re S_u(z) - \Re S_u(w) <0$. To this aim, observe first that the real part of the function $S_u$ is constant and equals zero on the unit circle $\mathbb {T}$. Identifying the complex plane with $\mathbb {R}^2$, the gradient of the real part of the function $S_u$ is thus orthogonal to $\mathbb {T}$. Its direction is given by the sign of the scalar product

$$\begin{aligned} \langle \nabla \Re S_u(z) , z \rangle _{\mathbb {R}^2} = zG'(z) -u. \end{aligned}$$

**Fig. 4:**
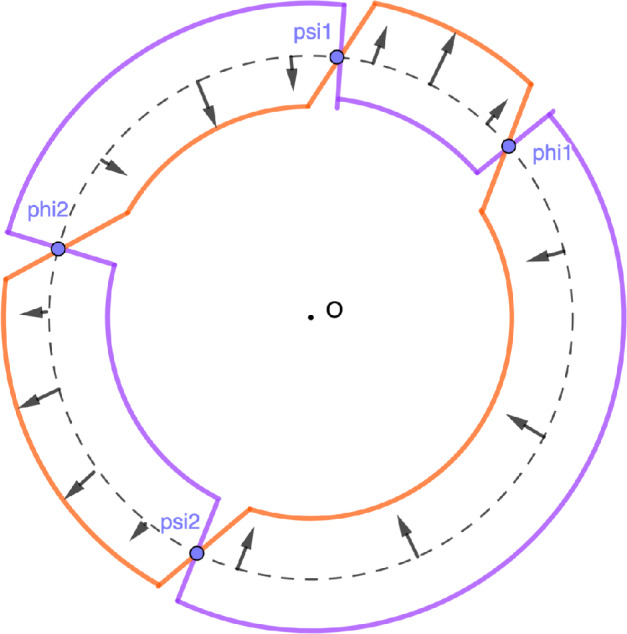
The variable *z* belongs to the purple contour $\gamma _u^<$, while *w* belongs to the orange one $\gamma _u^>$

Recall that for $u \in [u_{\min },u_{\max }]$, the set $\{ z \in \mathbb {T}, \hspace{0.1cm} zG'(z)\ge u \}$ consists of $L=L(u)$ arcs $[e^{i\phi _k(u)}, e^{i\psi _k(u)} ]$, $k=1,\dots ,L(u)$, and observe that the numbers $e^{i\phi _k(u)}$, $e^{i\psi _k(u)}$ are the critical points of the function $\Re S_u$. One deforms the circle $\mathbb {T}$ into a contour $\gamma _u^>$ by following the direction of the gradient $\nabla \Re S_u$, and into another contour $\gamma _u^{<}$ by following the opposite direction of the gradient $\nabla \Re S_u$, see Figure 4. By construction, we have

$$\begin{aligned} \Re S_u(z) \ge 0, \quad z \in \gamma _u^>, \end{aligned}$$

and

$$\begin{aligned} \Re S_u(z) \le 0, \quad z \in \gamma _u^<, \end{aligned}$$

with equality only at the critical points $e^{i\phi _k(u)}$, $e^{i\psi _k(u)}$, $k=1,\dots ,L(u)$. By the preceding discussion, the dominated convergence theorem implies that

32 $$\begin{aligned} \lim _{\alpha \rightarrow + \infty } \exp \left( \alpha ^\delta \right) \int _{z \in \gamma _u^<} \int _{w \in \gamma _u^>} \frac{\exp \left( \alpha \left( S_u(z)-S_u(w)\right) \right) }{z-w} \frac{dzdw}{z^{x}w^{-y+1}} =0 \end{aligned}$$

for all $u \in [u_{\min },u_{\max }]$, all $\delta \in (0,1)$ and all $x,y \in \mathbb {Z}$. Observe now that the residue at $z=w$ integrated in the expression (31) is no longer integrated in (32) on the arcs $[e^{i\phi _k(u)},e^{i\psi _k(u)}]$. Since this residue is $w^{y-x-1}$, we have from (32)

$$\begin{aligned}  &   K_\alpha (x+ \alpha u, y + \alpha u) \\  &   \quad = \frac{1}{(2i\pi )^2}\int _{z \in \gamma _u^<} \int _{w \in \gamma _u^>} \frac{\exp \left( \alpha \left( S_u(z)-S_u(w)\right) \right) }{z-w} \frac{dzdw}{z^{x}w^{-y+1}} + \sum _{k=1}^{L(u)}\frac{1}{2\pi } \int _{\phi _k(u)}^{\psi _k(u)} e^{i\theta (y-x)}d\theta \\  &   \quad =O\left( \exp \left( -\alpha ^\delta \right) \right) + \mathcal {S}(u)(x,y). \end{aligned}$$

Since $[u_{\min },u_{\max }]$ is compact, the $O\left( \exp \left( -\alpha ^\delta \right) \right)$ is uniform in *u*. Recalling Definition (19), we obtain (11) and the proof is complete.

### A Remark on Shift Invariant Subspaces

#### Wiener-Helson’s Theorem on the Classification of Shift Invariant Subspaces

We here give a classification of shift invariant subspaces of $\ell ^2(\mathbb {Z})$ due to Wiener and Helson (Theorem 3.3). The material we present is quite standard and may be found, for instance, in the first pages of the book [19].

Let $S : \ell ^2(\mathbb {Z}) \rightarrow \ell ^2(\mathbb {Z})$ be the shift

$$\begin{aligned} S (a_k)_{k \in \mathbb {Z}} = (a_{k-1})_{k \in \mathbb {Z}}, \quad (a_k)_{k \in \mathbb {Z}} \in \ell ^2(\mathbb {Z}). \end{aligned}$$

We say that a closed subspace $\mathcal {E} \subset \ell ^2(\mathbb {Z})$ is shift invariant if $S(\mathcal {E}) \subset \mathcal {E}$. If $\mathcal {E}$ is shift invariant, it is said to be *simply* invariant if $S(\mathcal {E}) \ne \mathcal {E}$ and *doubly* invariant otherwise.

We equip the unit circle $\mathbb {T}$ with the normalized Lebesgue measure and recall the Fourier-Plancherel unitary isomorphism

$$\begin{aligned} \mathcal {F} : L^2(\mathbb {T})&\rightarrow \ell ^2(\mathbb {Z}) \\ F&\mapsto (\hat{F}(k))_{k \in \mathbb {Z}}, \end{aligned}$$

where

$$\begin{aligned} \hat{F}(k):= \frac{1}{2\pi }\int _{-\pi }^\pi e^{-i\theta k} F(e^{i\theta }) d\theta = \frac{1}{2i \pi } \int _{|z|=1} z^{-k} F(z) \frac{dz}{z}. \end{aligned}$$

The map *S* is conjugated to the multiplication by *z* by the Fourier-Plancherel isomorphism.

The Hardy space $H^2(\mathbb {T})$ is defined as the closed linear space of functions of $L^2(\mathbb {T})$ with vanishing negative Fourier coefficients: $H^2(\mathbb {T}):= \{ F \in L^2(\mathbb {T}), \hspace{0.1cm} \hat{F}(k)=0, \hspace{0.1cm} k=-1,-2,\dots \}$. Before stating the Wiener-Helson’s theorem classifying the subspaces of $\ell ^2(\mathbb {Z})$ which are invariant by the shift *S* (or, equivalently, the subspaces of $L^2(\mathbb {T})$ which are invariant by the multiplication by *z*), we make the following elementary observation: if a function $F \in L^2(\mathbb {T})$ is such that $|F(z)|=1$ for almost all $z \in \mathbb {T}$, then the functions $z \mapsto F(z)z^n$, $n=0,1,\dots$ form an orthonormal basis of $FH^2(\mathbb {T})$, and the space $FH^2(\mathbb {T})$ is invariant by the multiplication by *z*. Let us write

$$\begin{aligned} \mathcal {E}_F:= \mathcal {F}(F H^2(\mathbb {T})). \end{aligned}$$

The space $\mathcal {E}_F$ is then a simply shift invariant subspace of $\ell ^2(\mathbb {Z})$. Helson’s theorem, which is the second point of the Theorem 3.3, establishes the reversed statement. The first point is due to Wiener, and classifies doubly shift invariant subspaces.

##### [Style2 Style2]Theorem 3.3

Let $\mathcal {E} \subset \ell ^2(\mathbb {Z})$ be a shift invariant subspace. Then we have the following

- If the subspace $\mathcal {E}$ is doubly invariant, then there exists a borel set $A \subset \mathbb {T}$ such that $\mathcal {E}= \mathcal {F} \left( \mathfrak {1}_A L^2(\mathbb {T}) \right)$, where $\mathfrak {1}_A$ is the indicator function of *A*.
- If the subspace $\mathcal {E}$ is simply invaraint, then there exists a function $F \in L^2(\mathbb {T})$ verifying $|F(z)|=1$ for almost every $z \in \mathbb {T}$ and such that $\mathcal {E}=\mathcal {F} \left( F H^2(\mathbb {T}) \right)$.

#### The Schur Measures Revisited

We now connect the preceding discussion with Schur measures and their limit processes. Let *G* be a function holomorphic in a neighborhood of the unit circle $\mathbb {T}$ such that $G(z) \in i \mathbb {R}$ for $z \in \mathbb {T}$, i.e., such that $\hat{G}(-k)=- \overline{ \hat{G}(k)}$ for all $k \in \mathbb {Z}$. We set

$$\begin{aligned} F(z) = \exp \left( G(z^{-1}) \right) . \end{aligned}$$

We have $|F(z)|=1$ for almost every $z \in \mathbb {T}$.

Let $K_F$ be the kernel of the orthogonal projection onto $\mathcal {E}_F$. By the Macchi-Soshnikov/Shirai-Takahashi theorem, the kernel $K_F$ serves as a correlation kernel of a determinantal point process. The following proposition says that the determinantal point process with kernel $K_F$ is a symmetric Schur measure, up to the transformation $X \mapsto -X$, $X \in \textrm{Conf}(\mathbb {Z})$.

##### Proposition 3.4

Let $\rho : \Lambda \rightarrow \mathbb {C}$ be the specialization defined by

$$\begin{aligned} \rho (p_k)= k \hat{G}(k), \quad k=1,2,\dots \end{aligned}$$

and let $\mathbb {P}_\rho$ be the symmetric Schur measure with specialization $\rho$. Let $K_\rho$ be the correlation kernel of the corresponding determinantal point process $\mathfrak {S}^* \mathbb {P}_\rho$ on $\mathbb {Z}$. Then, for all $x, y \in \mathbb {Z}$, we have

33 $$\begin{aligned} K_F(x,y) = K_\rho (-x,-y). \end{aligned}$$

##### Proof

Since the family $\{ (\hat{F}(k-n))_{k \in \mathbb {Z}}, \hspace{0.1cm} n=0,1,\dots \}$ is an orthonormal basis of $\mathcal {E}_F$, we have

$$\begin{aligned} K_F(x,y)&= \sum _{n =0}^{+ \infty } \hat{F}(x-n) \overline{\hat{F}(y-n)} \\&= \sum _{n=0}^{+ \infty } \frac{1}{(2 \pi )^2} \int _{-\pi }^\pi e^{-i\theta (x-n)} F(e^{i\theta }) d\theta \int _{-\pi }^\pi e^{i\theta '(y-n)} F^{-1}(e^{i\theta '})d\theta ', \end{aligned}$$

where we used the fact that $|F(z)|=1$ for $z \in \mathbb {T}$. Changing the variable $\theta \mapsto -\theta$, $\theta ' \mapsto -\theta '$, we obtain

$$\begin{aligned} K_F(x,y)&= \sum _{n = 0}^{+ \infty } \frac{1}{(2\pi )^2} \int _{-\pi }^\pi e^{i \theta (x-n)} F(e^{-i \theta }) d\theta \int _{- \pi }^\pi e^{i\theta '(n-y)} F^{-1}(e^{-i \theta '}) d\theta ' \\&= \sum _{n = 0}^{+ \infty } \frac{1}{(2i \pi )^2} \int _{|z|=1} z^{x-n} F(z^{-1}) \frac{dz}{z} \int _{|w|=1} w^{n-y} F^{-1}(w^{-1}) \frac{dw}{w}. \end{aligned}$$

Since *F* is holomorphic in a neighborhood of $\mathbb {T}$, we have for $\varepsilon >0$ small enough that

$$\begin{aligned} K_F(x,y)&= \sum _{n = 0}^{+ \infty } \frac{1}{(2i \pi )^2} \int _{|z|=1+ \varepsilon } z^{x-n} F(z^{-1}) \frac{dz}{z} \int _{|w|=1- \varepsilon } w^{n-y} F^{-1}(w^{-1}) \frac{dw}{w} \\&= \frac{1}{(2 i \pi )^2} \int _{|z|=1+ \varepsilon } \int _{|w|=1-\varepsilon } \frac{F(z^{-1})F^{-1}(w^{-1})}{1-w/z} \frac{dzdw}{z^{-x+1}w^{y+1}} \\&=\frac{1}{(2 i \pi )^2} \int _{|z|=1+ \varepsilon } \int _{|w|=1-\varepsilon } \frac{F(z^{-1})F^{-1}(w^{-1})}{z-w} \frac{dzdw}{z^{-x}w^{y+1}}, \end{aligned}$$

where we used Fubini’s theorem for the second line. Observing that from the definitions of the function *F* and of the specialization $\rho$, we have

$$\begin{aligned} F(z^{-1})F^{-1}(w^{-1})&=\exp \left( G(z)-G(w) \right) \\&=\exp \left( \sum _{k \ge 1} \hat{G}(k)(z^{k} -w^k) +\overline{\hat{G}(k)}(w^{-k}-z^{-k} ) \right) \\&= \exp \left( \sum _{k \ge 1} \frac{\rho (p_k)}{k}(z^k-w^{k}) + \frac{\overline{\rho (p_k)}}{k}(w^{-k} - z^{-k}) \right) , \end{aligned}$$

and recalling Formula (7) for the kernel $K_\rho$, the proof is complete. $\square$

##### Remark 3.5

As observed in [8], Proposition 3.4 remains true for more general symmetric Schur measures, i.e., when the function *G* is not necessarily holomorphic in a neighborhood of $\mathbb {T}$. We chose to restrict ourselves to the case when *G* is holomorphic in a neighborhood of $\mathbb {T}$ in order to enlight the interpretation of Proposition 1.2 in terms of shift invariant subspaces, which we describe below.

#### An Interpretation of Proposition 1.2

Observe that the limit kernels $\mathcal {S}(u)$ are projection kernels onto doubly shift invariant subspaces

$$\begin{aligned} \mathcal {E}(u):= \mathcal {F} \left( \mathfrak {1}_{\sqcup [e^{i\phi _k(u)},e^{i\psi _k(u)}] } L^2(\mathbb {T}) \right) , \end{aligned}$$

since we have

$$\begin{aligned} \mathcal {S}(u)(x,y)= \sum _{k=1}^{L(u)} \hat{\mathfrak {1}}_{[e^{i\phi _k(u)},e^{i\psi _k(u)}]}(x-y). \end{aligned}$$

Proposition 1.2, and more precisely the convergence (30) establishes thus a link between simply and doubly shift invariant subspaces of $\ell ^2(\mathbb {Z})$. In a certain regime and in a certain sense, some simply invariant subspaces converge to doubly invariant subspaces. We make this fact precise: if *G* is a holomorphic function in the neighborhood of $\mathbb {T}$ as above, we can define a function $F_\alpha (z)= \exp \left( \alpha G(z^{-1}) \right)$, and consider the simply invariant subspace $\mathcal {E}_{F_\alpha }$. With a little computation, we have that the kernel

$$\begin{aligned}(x,y) \mapsto K_{F_\alpha }(\lfloor \alpha u \rfloor + x, \lfloor \alpha u \rfloor +y) \end{aligned}$$

is the kernel of the orthogonal projection onto the subspace $S^{-\lfloor \alpha u \rfloor } (\mathcal {E}_{F_\alpha })$. By Proposition 3.4 and slightly adapting the proof of the convergence (30), we have that, as $\alpha \rightarrow + \infty$, the space $S^{-\lfloor \alpha u \rfloor } (\mathcal {E}_{F_\alpha })$ converges to $\mathcal {E}(u)$ in the sense that the orthogonal projection onto $S^{-\lfloor \alpha u \rfloor } (\mathcal {E}_{F_\alpha })$ converges to the orthogonal projection onto $\mathcal {E}(u)$ in the strong operator topology.

### Proof of Lemmas 3.1 and 3.2

#### Proof of Lemma 3.1

By Proposition 2.2 and since the kernel $K_\alpha$ is symmetric, it suffices to prove that for all $x,y \in \mathbb {Z}$, there exists $C>0$ such that

34 $$\begin{aligned} |K_\alpha (x+\alpha u_1,y+ \alpha u_2) | \le \frac{C}{\alpha | u_1-u_2|} \end{aligned}$$

for all $u_1,u_2 \in [u_{\min },u_{\max }]$, $u_1 \ne u_2$ and all $\alpha >0$ sufficiently large. As in the proof of Proposition 1.2, write

$$\begin{aligned} K_\alpha (x+ \alpha u_1, y+ \alpha u_2) = \frac{1}{(2i \pi )^2} \int _{|z|=1 + \varepsilon } \int _{|w|=1- \varepsilon } \frac{\exp \left( S_{u_1}(z) - S_{u_2}(w) \right) }{z-w} \frac{dzdw}{z^{x}w^{-y+1}}. \end{aligned}$$

**Fig. 5:**
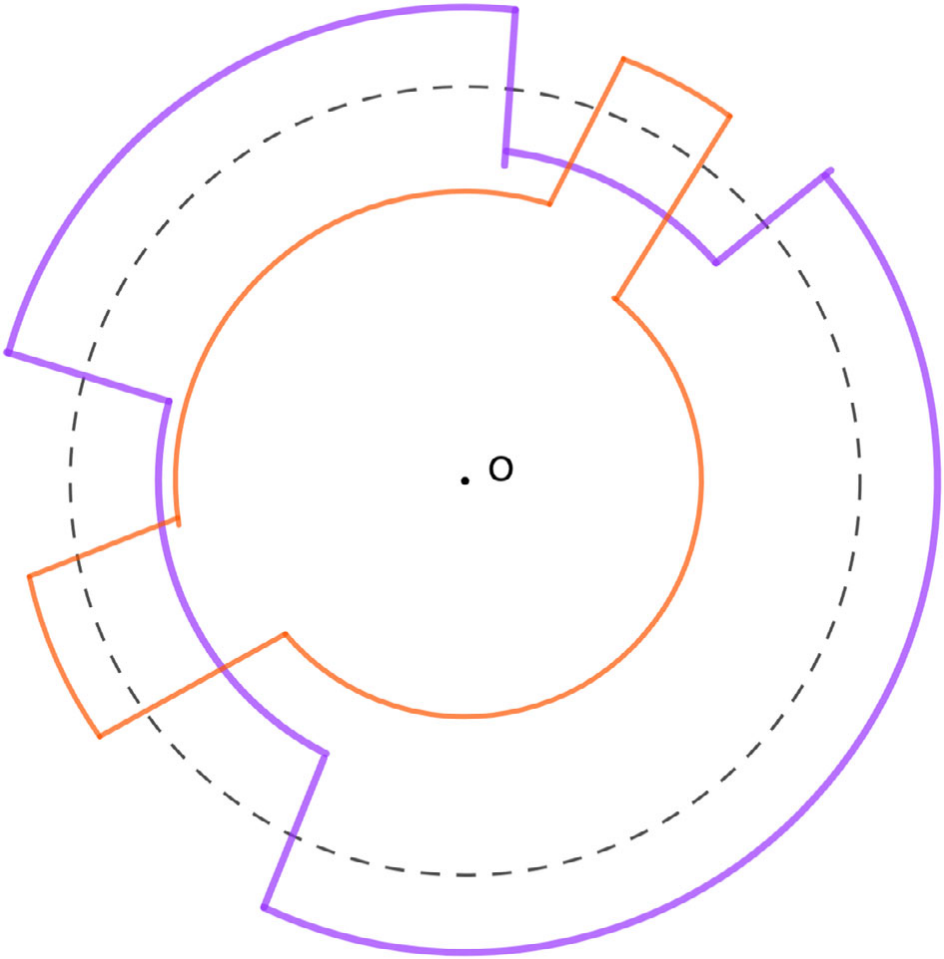
The contours $\gamma _{u_1}^<$ and $\gamma _{u_2}^>$

We then deform the contour and integrate over $z \in \gamma _{u_1}^<$ and $w \in \gamma _{u_2}^>$ so that

$$\begin{aligned} \Re S_{u_1}(z) - \Re S_{u_2}(w) <0, \end{aligned}$$

except at a finite number of points (see Figure 5). In order to recover the value of the kernel $K_\alpha$, we must again integrate the residue at $z=w$ over a finite number of arcs $[e^{i\phi _k},e^{i\psi _k}]$ which depend on $u_1$ and $u_2$. We have

$$\begin{aligned} K_\alpha (x+ \alpha u_1, y+ \alpha u_2) = \frac{1}{(2i \pi )^2} \int _{ z \in \gamma _{u_1}^<} \int _{w \in \gamma _{u_2}^>} \cdots + \sum _{k} \frac{1}{2 \pi } \int _{\phi _k}^{\psi _k} e^{i \theta (\alpha u_2 - \alpha u_1 + y -x )} d\theta . \end{aligned}$$

By construction, the integral

$$\begin{aligned} \int _{ z \in \gamma _{u_1}^<} \int _{w \in \gamma _{u_2}^>} \cdots \end{aligned}$$

is dominated by $\exp (-\alpha ^\delta )$, for any $\delta \in (0,1)$. By a direct integration, we have

$$\begin{aligned} \left| \int _{\phi _k}^{\psi _k} e^{i \theta (\alpha u_2 - \alpha u_1 + y -x )} d\theta \right| \le \frac{C}{\alpha |u_1-u_2|} \end{aligned}$$

for all $\alpha >0$ sufficiently large and all $u_1 \ne u_2$, where *C* only depens on *x* and *y*. We have just established (34), and the lemma is proved.

#### Proof of Lemma 3.2

Since the points $e^{i\phi _k(u)}$, $e^{i\psi _k(u)}$ are the solutions of the equation $zG'(z)=u$, the function

$$\begin{aligned}u \mapsto \mathcal {S}(u)(x,y) \end{aligned}$$

is continuous in *u* for any fixed $x,y \in \mathbb {Z}$. Since $[u_{\min },u_{\max }]$ is compact, this established the proof of Lemma 3.2.

## Proof of Theorem 1.8

### Control of the Covariances

Let $r>0$ and let $\mathbb {P}_r$ be the image by $\mathfrak {S}_2$ of the Schur process $\tilde{\mathbb {P}}_{e^{-r}}$ defined in section 1.3. We start by stating the lemmas we use for the control of the covariances for $\mathbb {P}_r$, distinguishing the situations where the considered positions are far away from each other or not.

#### Lemma 4.1

Let $\mathcal {K} \subset \mathbb {R}^2$ be a compact set, and let $m \subset \mathbb {E}$ be finite. Let $\overline{m}$ denote the supremum norm of *m*

$$\begin{aligned} \overline{m}=\max \{ |t|,|h|, \hspace{0.1cm} (t,h) \in m \}. \end{aligned}$$

Then for any $\delta \in (0,1)$, there exists *C* which only depends on $\delta$, $\mathcal {K}$ and *m*, such that for all $r >0$ sufficiently small and any $(\tau _1,\chi _1),(\tau _2,\chi _2) \in A\cap \mathcal {K}$ such that

35 $$\begin{aligned} \max \{ |\tau _1-\tau _2|, |\chi _1-\chi _2| \} > \overline{m}r, \end{aligned}$$

one has

$$\begin{aligned} \left| \mathbb {E}_r \left[ c_{\frac{1}{r}(\tau _1,\chi _1)+m}c_{\frac{1}{r}(\tau _2,\chi _2)+m} \right] -\mathbb {E}_r \left[ c_{\frac{1}{r}(\tau _1,\chi _1)+m}\right] \mathbb {E}_r\left[ c_{\frac{1}{r}(\tau _2,\chi _2)+m} \right] \right| \le \frac{C\exp \left( -r^{-\delta }\right) }{|\tau _1-\tau _2|^2} \end{aligned}$$

when $$\tau _1 \ne \tau _2$$, and

$$\begin{aligned} \left| \mathbb {E}_r \left[ c_{\frac{1}{r}(\tau ,\chi _1)+m}c_{\frac{1}{r}(\tau ,\chi _2)+m} \right] -\mathbb {E}_r \left[ c_{\frac{1}{r}(\tau ,\chi _1)+m}\right] \mathbb {E}_r\left[ c_{\frac{1}{r}(\tau ,\chi _2)+m} \right] \right| \le \frac{Cr}{|\chi _1-\chi _2|} \end{aligned}$$

when $$\tau _1=\tau _2=\tau $$.

The next lemma we need is obtained as a simple corollary of Proposition 4.3.

#### Lemma 4.2

For any compact $\mathcal {K} \subset \mathbb {R}$ and any finite subsets $m,m' \subset E$, there exists *C* such that for any $(\tau ,\chi ) \in A\cap \mathcal {K}$, and any sufficiently small $r>0$, we have

$$\begin{aligned} \left| \mathbb {E}_r \left[ c_{\frac{1}{r}(\tau ,\chi )+m} c_{\frac{1}{r}(\tau ,\chi )+m'}\right] - \mathbb {E}_r \left[ c_{\frac{1}{r}(\tau ,\chi )+m}\right] \mathbb {E}_r \left[ c_{\frac{1}{r}(\tau ,\chi )+m'} \right] \right| \le C. \end{aligned}$$

### Proof of Theorem 1.8

Let $\mathcal {K} \subset \mathbb {R}^2$ be a compact set containing the support of *f*. We denote by $A_{r,\mathcal {K}}$ the set

$$\begin{aligned} A_{r,\mathcal {K}}=r^{-1}(A \cap \mathcal {K} ) \cap E. \end{aligned}$$

By Proposition 1.6, there exists $C>0$ such that

$$\begin{aligned} \left| \mathbb {E}_r \Sigma ( f,m,r) - r^2 \sum _{(t,h) \in A_{r,\mathcal {K}}} f(rt,rh) \mathbb {E}_{(rt,rh)} \left[ c_{m} \right] \right| \le C |A_{r,\mathcal {K}}| r^{3}, \end{aligned}$$

where $|A_{r,\mathcal {K}}|$ is the cardinality of $A_{r,\mathcal {K}}$. We first remark that

36 $$\begin{aligned} |A_{r,\mathcal {K}}| = O \left( r^{-2} \right) , \end{aligned}$$

which implies

$$\begin{aligned} \mathbb {E}_{r} \Sigma (f,m,r)= r^2 \sum _{(t,h) \in A_{r,\mathcal {K}}} f(rt,rh) \mathbb {E}_{(rt,rh)} \left[ c_{m}\right] + O(r). \end{aligned}$$

Observing then that

$$\begin{aligned} r^2 \sum _{(t,h) \in A_{r,\mathcal {K}}} f(rt,rh) \mathbb {E}_{(rt,rh)} \left[ c_{m}\right] \end{aligned}$$

is a Riemann sum for the integral *I*(*f*, *m*), we obtain that

$$\begin{aligned} \lim _{r \rightarrow 0} \mathbb {E}_{r} \Sigma (f,m,r) = I(f,m). \end{aligned}$$

By the Chebyshev inequality, it suffices now to prove that

37 $$\begin{aligned} \text {Var}_{r} \left( \Sigma (f,m,r) \right) \rightarrow 0 \end{aligned}$$

as *r* tends to 0, where

38 $$\begin{aligned} \text {Var}_{r} \left( \Sigma (f,m,r) \right)= &   \mathbb {E}_r \left[ \left( \Sigma (f,m,r) - \mathbb {E}_r \Sigma (f,m,r) \right) ^2 \right] \nonumber \\= &   r^4 \sum _{(t_1h_1),(t_2,h_2) \in A_{r,\mathcal {K}}} f(rt_1,rh_1)f(rt_2,rh_2) \nonumber \\  &   \times \left( \mathbb {E}_r [ c_{(t_1,h_1)+m}c_{(t_2,h_2)+m} ] - \mathbb {E}_r [ c_{(t_1,h_1)+m} ] \mathbb {E}_r [ c_{(t_2,h_2)+m} ] \right) . \nonumber \\ \end{aligned}$$

We set $\overline{m}=\max \{ |t|,|h|, \hspace{0.1cm} (t,h) \in m \}$, and we partition $A_{r,\mathcal {K}}^2$ into three sets

$$\begin{aligned} A_{r,\mathcal {K}}^2=A_{r,\mathcal {K}}^{>} \sqcup A_{r,\mathcal {K}}^{>=} \sqcup A_{r,\mathcal {K}}^{\le }, \end{aligned}$$

where :

$$\begin{aligned} A_{r,\mathcal {K}}^{>}&=\left\{ ((t_1,h_1), (t_2,h_2)) \in A_{r,\mathcal {K}}^2, \hspace{0.1cm} \max \{ |t_1-t_2|, |h_1-h_2|\}> \overline{m}, \hspace{0.1cm} t_1 \ne t_2 \right\} , \\ A_{r,\mathcal {K}}^{>=}&= \left\{ ((t,h_1), (t,h_2)) \in A_{r,\mathcal {K}}^2, \hspace{0.1cm} |h_1-h_2|> \overline{m} \right\} ,\\ A_{r,\mathcal {K}}^{\le }&= A_{r,\mathcal {K}}^2 \setminus \left( A_{r,\mathcal {K}}^{>} \sqcup A_{r,\mathcal {K}}^{>=} \right) \\&= \left\{ ((t_1,h_1),(t_2,h_2)) \in A_{r,\mathcal {K}}^2, \hspace{0.1cm} \max \{ |t_1-t_2|, |h_1-h_2|\} \le \overline{m} \right\} . \end{aligned}$$

We first estimate the variance (38) by

39 $$\begin{aligned} \text {Var}_{r} \left( \Sigma (f,m,r) \right)\le &   C r^4 \left( \sum _{\left( (t_1h_1),(t_2,h_2)\right) \in A_{r,\mathcal {K}}^>} \left| \mathbb {E}_r [ c_{(t_1,h_1)+m}c_{(t_2,h_2)+m} ] \right. \right. \nonumber \\  &   \left. \left. - \mathbb {E}_r [ c_{(t_1,h_1)+m} ] \mathbb {E}_r [ c_{(t_2,h_2)+m} ] \right| \right. \nonumber \\  &   \left. + \sum _{ \left( (t,h_1),(t,h_2)\right) \in A_{r,\mathcal {K}}^{>=} } \left| \mathbb {E}_r [ c_{(t,h_1)+m}c_{(t,h_2)+m} ] \right. \right. \nonumber \\  &   \left. \left. - \mathbb {E}_r [ c_{(t,h_1)+m} ] \mathbb {E}_r [ c_{(t,h_2)+m} ] \right| \right. \nonumber \\  &   \left. + \sum _{\left( (t_1h_1),(t_2,h_2)\right) \in A_{r,\mathcal {K}}^{\le }} \left| \mathbb {E}_r [ c_{(t_1,h_1)+m}c_{(t_2,h_2)+m} ] \right. \right. \nonumber \\  &   \left. \left. - \mathbb {E}_r [ c_{(t_1,h_1)+m} ] \mathbb {E}_r [ c_{(t_2,h_2)+m} ] \right| \right) , \end{aligned}$$

where *C* only depends on *f*. Let $(t_1,h_1),(t_2,h_2) \in A_{r,\mathcal {K}}$. By definition, there exists $(\tau _1,\chi _1),(\tau _2,\chi _2) \in A \cap \mathcal {K} \cap rE$ such that :

$$\begin{aligned} (t_1,h_1)=\frac{1}{r}(\tau _1,\chi _1), \hspace{0.1cm} (t_2,h_2) =\frac{1}{r}(\tau _2,\chi _2). \end{aligned}$$

We first consider the case when $\left( (t_1,h_1),(t_2,h_2)\right) \in A_{r,\mathcal {K}}^>$. The corresponding points $(\tau _1,\chi _1),(\tau _2,\chi _2)$ satisfy condition (35), and thus by Lemma 4.1, we have in particular the estimate

$$\begin{aligned} \left| \mathbb {E}_r \left[ c_{\frac{1}{r}(\tau _1,\chi _1)+m}c_{\frac{1}{r}(\tau _2,\chi _2)+m} \right] -\mathbb {E}_r \left[ c_{\frac{1}{r}(\tau _1,\chi _1)+m}\right] \mathbb {E}_r\left[ c_{\frac{1}{r}(\tau _2,\chi _2)+m} \right] \right| \le Cr, \end{aligned}$$

where *C* is uniform. Since

$$\begin{aligned} \left| A_{r,\mathcal {K}}^> \right| = O \left( r^{-4} \right) , \end{aligned}$$

we obtain that

40 $$\begin{aligned} \sum _{\left( (t_1h_1),(t_2,h_2)\right) \in A_{r,\mathcal {K}}^>} \left| \mathbb {E}_r [ c_{(t_1,h_1)+m}c_{(t_2,h_2)+m} ] - \mathbb {E}_r [ c_{(t_1,h_1)+m} ] \mathbb {E}_r [ c_{(t_2,h_2)+m} ] \right| \le Cr^{-3} \end{aligned}$$

where *C* only depends on $\mathcal {K}$ and *m*.

In the case when $\left( (t_1,h_1,),(t_2,h_2)\right) \in A_{r,\mathcal {K}}^{>=}$, we have by Lemma 4.1 that

$$\begin{aligned} \left| \mathbb {E}_r \left[ c_{\frac{1}{r}(\tau _1,\chi _1)+m}c_{\frac{1}{r}(\tau _2,\chi _2)+m} \right] -\mathbb {E}_r \left[ c_{\frac{1}{r}(\tau _1,\chi _1)+m}\right] \mathbb {E}_r\left[ c_{\frac{1}{r}(\tau _2,\chi _2)+m} \right] \right| \le C \end{aligned}$$

where *C* is uniform. Since

$$\begin{aligned} |A_{r,\mathcal {K}}^{>=}| = O \left( r^{-3} \right) , \end{aligned}$$

we have

41 $$\begin{aligned} \sum _{(t,h_1),(t,h_2) \in A_{r,\mathcal {K}}^{>=}} \left| \mathbb {E}_r [ c_{(t,h_1)+m}c_{(t,h_2)+m} ] - \mathbb {E}_r [ c_{(t,h_1)+m} ] \mathbb {E}_r [ c_{(t,h_2)+m} ] \right| \le Cr^{-3}, \end{aligned}$$

where *C* only depends on $\mathcal {K}$ and *m*.

When $\left( (t_1,h_1),(t_2;h_2)\right) \in A_{r,\mathcal {K}}^{\le }$, there exists finite subsets $m',m'' \subset E$ and $(\tau ,\chi ) \in A\cap K$ such that

$$\begin{aligned} (t_1,h_1)+m= \frac{1}{r}(\tau ,\chi ) +m', \hspace{0.1cm} (t_2,h_2)+m = \frac{1}{r}(\tau ,\chi ) +m''. \end{aligned}$$

Observe that there is only a finite number of possible sets $m'$ and $m''$. Thus, by Lemma 4.2, we have

$$\begin{aligned} \left| \mathbb {E}_r \left[ c_{\frac{1}{r}(\tau ,\chi )+m'} c_{\frac{1}{r}(\tau ,\chi )+m''}\right] - \mathbb {E}_r \left[ c_{\frac{1}{r}(\tau ,\chi )+m'}\right] \mathbb {E}_r \left[ c_{\frac{1}{r}(\tau ,\chi )+m''} \right] \right| \le C \end{aligned}$$

where *C* is uniform. Since

$$\begin{aligned} |A_{r,\mathcal {K}}^{\le } |= O \left( r^{-2} \right) , \end{aligned}$$

we have

42 $$\begin{aligned} \sum _{(t,h_1),(t,h_2) \in A_{r,\mathcal {K}}^{\le } }\left| \mathbb {E}_r [ c_{(t,h_1)+m}c_{(t,h_2)+m} ] - \mathbb {E}_r [ c_{(t,h_1)+m} ] \mathbb {E}_r [ c_{(t,h_2)+m} ] \right| \le Cr^{-2}. \end{aligned}$$

Recalling the estimation (39), the inequalities (40), (41) and (42) establish (37). Theorem 1.8 is proved, assuming Proposition 1.6 and Lemmas 4.1 and 4.2.

### Proof of Proposition 1.6

We here follow the proof of [22], giving the error terms in the asymptotics we use. We define the dilogarithm function as being the analytic continuation of the series

$$\begin{aligned} \text {dilog}(1-z)=\sum _{n \ge 1} \frac{z^n}{n^2}, \quad |z|<1, \end{aligned}$$

with a cut along the half-line $(1,+\infty )$. We first prove that

43 $$\begin{aligned} -\log (z,q)_\infty = r^{-1}\text {dilog}(1-z) + O(1). \end{aligned}$$

as $q=e^{-r}$ tends to $1^-$. Indeed, we have

$$\begin{aligned} \log (z,q)_{\infty }&=\sum _{k \ge 0} \log (1-zq^k) = -\sum _{k \ge 0} \sum _{n \ge 1} \frac{z^nq^{nk}}{n} \\&=-\sum _{n \ge 1} \frac{z^n}{n} \sum _{k \ge 0} q^{nk} =-\sum _{n \ge 0} \frac{z^n}{n} \frac{1}{1-q^n}. \end{aligned}$$

With $q=e^{-r}$, we have

$$\begin{aligned} \frac{r}{1-e^{-nr}}=\frac{1}{n -n^2r + ...}=\frac{1}{n}(1+nr+...), \end{aligned}$$

and thus

$$\begin{aligned} \left| \frac{z^n}{n}\left( \frac{r}{1-e^{-nr}}- \frac{1}{n}\right) \right| \le r|z|^n, \end{aligned}$$

which establishes (43).

Let $\mathcal {K} \subset \mathbb {R}^2$ be compact and let $(\tau ,\chi ) \in A\cap \mathcal {K}$. We assume that $\tau \ge 0$ (see 4.4.1 below for the case $\tau \le 0$). We introduce the function

$$\begin{aligned} S(z;\tau ,\chi )=-(\tau /2+ \chi )\log (z)-\text {dilog}(1-1/z)+ \text {dilog}(1-e^{-\tau }z), \end{aligned}$$

and denote by $\gamma _\tau$ the circle

$$\begin{aligned} \gamma _\tau = \{ z \in \mathbb {C}, \hspace{0.1cm} |z|=e^{\tau /2} \}. \end{aligned}$$

By the estimate (43) and formula (13), we have that, for all *z* and *w* sufficiently closed to $\gamma _\tau$

44 $$\begin{aligned}  &   \displaystyle \left| \frac{\Phi (\tau /r+ t_1,z)}{\Phi (\tau /r+t_2,w)}\frac{1}{z^{\chi /r+h_1+\tau /2r+(t_1+1)/2}w^{-\chi /r-h_2-\tau /2r-(t_2+1)/2}} \right| \nonumber \\  &   \quad = \exp \left( \frac{1}{r} \left( \mathfrak {R} S(z;\tau ,\chi )-\mathfrak {R}S(w;\tau ,\chi ) \right) + O(1) \right) , \end{aligned}$$

where the *O*(1) term only depens on $\mathcal {K}$, $(t_1,h_1)$ and $(t_2,h_2)$. An observation made in [22] states that the real part of *S* on the circle $\gamma _\tau$ is constant, namely

45 $$\begin{aligned} \mathfrak {R}S(z;\tau ,\chi )=-\frac{\tau }{2}(\tau /2+\chi ),\quad z \in \gamma _\tau . \end{aligned}$$

**Fig. 6:**
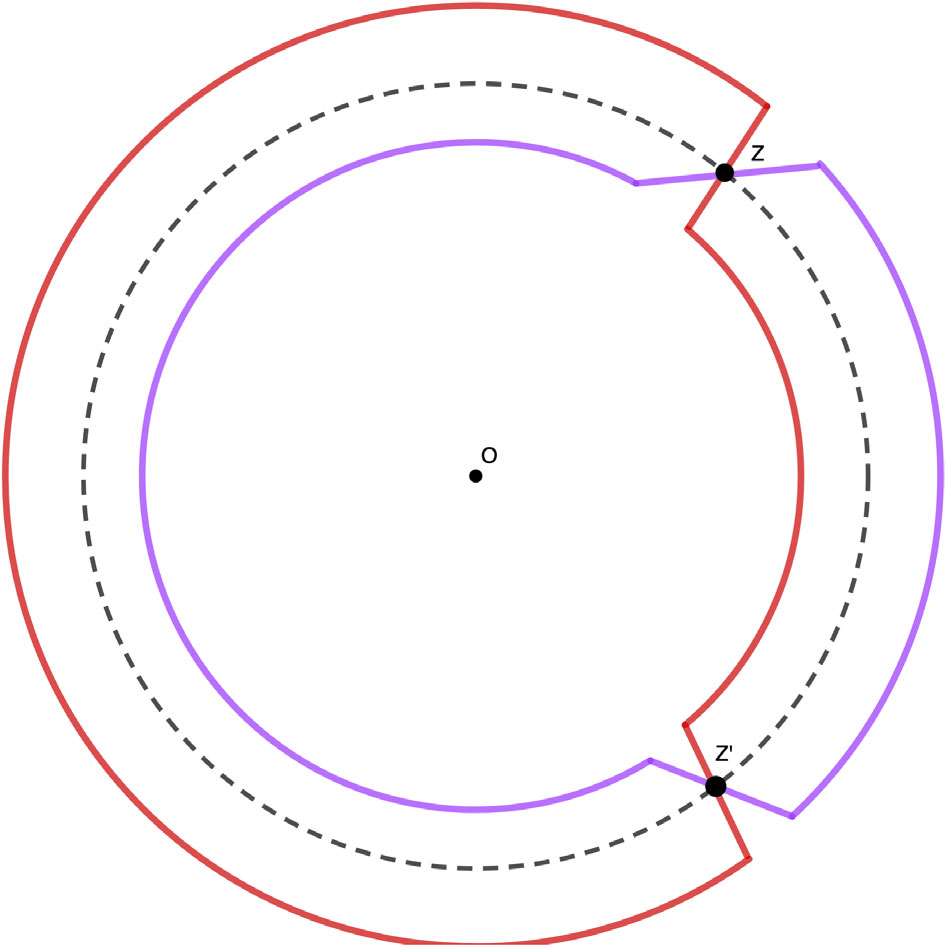
The contours $\gamma _\tau ^>$ and $\gamma _\tau ^<$ are the thick contours and the circle $\gamma _\tau$ is the dotted circle

It is also shown in [22] that, since $(\tau ,\chi ) \in A$, the function *S* has two distinct critical points on $\gamma _\tau$ : $e^{\tau }z(\tau ,\chi )$ and its complex conjugate. The computation of the gradient of the real part of *S* on $\gamma _\tau$ lead then the authors of [22] to deform the circle $\gamma _\tau$ into simple contours $\gamma _\tau ^>$ and $\gamma _\tau ^<$, both crossing the two critical points and verifying

46 $$\begin{aligned} \begin{aligned} z \in \gamma _\tau ^> \Rightarrow \mathfrak {R}S(z;\tau ,\chi ) \ge -\frac{\tau }{2}(\tau /2+\chi ), \\ z \in \gamma _\tau ^< \Rightarrow \mathfrak {R}S(z;\tau ,\chi ) \le -\frac{\tau }{2}(\tau /2+\chi ), \end{aligned} \end{aligned}$$

with equality only for $z\in \left\{ e^\tau z(\tau ,\chi ), e^\tau \overline{z(\tau ,\chi )} \right\}$ (see Figure 6). These simple facts imply that the integral

47 $$\begin{aligned} \int _{z \in \gamma _\tau ^<} \int _{w \in \gamma _\tau ^>} \exp \left( \frac{1}{r} \left( S(z;\tau ,\chi )-S(w;\tau ,\chi ) \right) \right) \frac{dzdw}{z-w} \end{aligned}$$

goes to zero as *r* tends to zero. Actually, the dominated convergence theorem implies that the integral (47) is $O\left( \exp \left( -r^{-\delta }\right) \right)$ for any $\delta \in (0,1)$.

Picking the residue at $z=w$, we arrive at

48 $$\begin{aligned}  &   \mathcal {K}_{e^{-r}} \left( \frac{\tau }{r} + t_1, \frac{\chi }{r} +h_1 ; \frac{\tau }{r} + t_2, \frac{\chi }{r} +h_2\right) \nonumber \\  &   \quad = \frac{1}{(2i\pi )^2} \int _{z \in \gamma _\tau ^<} \int _{w \in \gamma _\tau ^>} \exp \left( \frac{1}{r} \left( S(z;\tau ,\chi )-S(w;\tau ,\chi ) \right) + O(1) \right) \frac{dzdw}{z-w} \nonumber \\  &   \quad + \frac{1}{2i\pi } \int _{e^\tau \overline{z(\tau ,\chi )}}^{e^\tau z(\tau ,\chi )} \frac{(q^{1/2+\tau /r+t_2}w;q)_\infty }{(q^{1/2+ \tau /r +t_1}w;q)_\infty } \frac{dw}{w^{h_1-h_2+(t_1-t_2)/2}}, \end{aligned}$$

where the path of integration for the second integral crosses the interval $(0,e^\tau )$ for $t_1\ge t_2$ and the half-line $(-\infty ,0)$ otherwise. By the preceding discussion, the first integral rapidly tends to zero. Observe now that, using similar techniques as in the proof of (43), we have

49 $$\begin{aligned} \frac{(q^{1/2+\tau /r+t_2}w;q)_\infty }{(q^{1/2+ \tau /r +t_1}w;q)_\infty } = (1+O(r))\left( 1-e^{-\tau }w\right) ^{t_1-t_2}, \end{aligned}$$

where the *O*(*r*) term only depends on $\mathcal {K}$, $t_1$ and $t_2$. Performing the change of variable $w \mapsto e^{-\tau }w$ in the second integral of (48), we arrive at

$$\begin{aligned}  &   \frac{1}{2i\pi } \int _{e^\tau \overline{z(\tau ,\chi )}}^{e^\tau z(\tau ,\chi )} \frac{(q^{1/2+\tau /r+t_2}w;q)_\infty }{(q^{1/2+ \tau /r +t_1}w;q)_\infty } \frac{dw}{w^{h_1-h_2+(t_1-t_2)/2}} \\  &   \quad = \left( 1 +O(r) \right) e^{-\tau (h_1-h_2-(t_1-t_2)/2)} \mathcal {S}_{z(\tau ,\chi ) }( t_1-t_2,h_1-h_2). \end{aligned}$$

The factor $e^{-\tau (h_1-h_2-(t_1-t_2)/2)}$ can be ignored (see Remark 2.1). Proposition 1.6 is proved.

### Proof of Lemmas 4.1 and 4.2

#### A Remark and a Proposition

For $\tau <0$, one has to replace the function *S* by

$$\begin{aligned} \tilde{S}(z,\tau ,\chi )=-(|\tau |/2+\chi )\log (z)-\text {dilog}(1-z)+\text {dilog}(1-e^{-|\tau |}/z). \end{aligned}$$

The function $\tilde{S}$ inherits the same properties as the function *S*: it is constant on the circle $\gamma _{|\tau |}$ and has two complex conjugated critical points on this circle provided $(\tau ,\chi ) \in A$. This is why we will only consider positve values of $\tau$ in the sequel.

The critical points of *S* are the roots of the quadratic polynomial

$$\begin{aligned} (1-1/z)(1-e^{-\tau }z)=e^{-\tau /2-\chi }. \end{aligned}$$

For this reason, we have the following proposition:

##### Proposition 4.3

For any fixed $(\Delta t, \Delta h) \in E$, the function :

$$\begin{aligned} (\tau ,\chi ) \mapsto \mathcal {S}_{z(\tau ,\chi )}(\Delta t, \Delta h) \end{aligned}$$

is continuous.

#### Proof of Lemma 4.1

Let $m \subset E$ be a pattern, of cardinality *l*, let $\mathcal {K} \subset \mathbb {R}^2$ be a compact set and let $(\tau _1,\chi _1), (\tau _2,\chi _2) \in A\cap \mathcal {K}$ be as in the statement of the lemma. The condition (35) implies that the sets $\frac{1}{r}(\tau _1,\chi _1)+m$ and $\frac{1}{r}(\tau _2,\chi _2)+ m$ are disjoint. By Proposition 2.2, the covariance

$$\begin{aligned} \textrm{cov}_r(c_{ \frac{1}{r}(\tau _1,\chi _1) + m}, c_{m \frac{1}{r} (\tau _2,\chi _2) + m})&= \mathbb {E}_r [c_{ \frac{1}{r}(\tau _1,\chi _1) + m} c_{m \frac{1}{r} (\tau _2,\chi _2) + m}] \\&\quad - \mathbb {E}_r [c_{ \frac{1}{r}(\tau _1,\chi _1) + m} ] \mathbb {E}_r [c_{m \frac{1}{r} (\tau _2,\chi _2) + m}] \end{aligned}$$

is a sum of $(2l)! - (l!)^2$ terms, each of them containing a factor of the form

50 $$\begin{aligned} \mathcal {K}_{e^{-r}}\left( \frac{1}{r}(\tau _1,\chi _1){+}m_{i_1}; \frac{1}{r}(\tau _2,\chi _2){+}m_{j_1}\right) \mathcal {K}_{e^{-r}}\left( \frac{1}{r}(\tau _2,\chi _2){+}m_{j_2}; \frac{1}{r}(\tau _1,\chi _1){+}m_{i_2}\right) . \end{aligned}$$

By Propositions 1.6 and 4.3, the other factors are bounded by a bound only depending on $\mathcal {K}$ and *m*. By similar methods as in the proof of Proposition 1.6, we will show that the product (50) is small.

The product (50) can be written as a quadruple integral

$$\begin{aligned}  &   \displaystyle \mathcal {K}_{e^{-r}}\left( \frac{1}{r}(\tau _1,\chi _1)+m_{i_1}; \frac{1}{r}(\tau _2,\chi _2){+}m_{j_1}\right) \mathcal {K}_{e^{-r}}\left( \frac{1}{r}(\tau _2,\chi _2){+}m_{j_2}; \frac{1}{r}(\tau _1,\chi _1){+}m_{i_2}\right) \\  &   \quad = \frac{1}{(2i\pi )^4} \int _{z \in (1+\varepsilon )\gamma _{\tau _1}} dz\int _{w \in (1-\varepsilon )\gamma _{\tau _2}} dw\int _{z' \in (1-\varepsilon )\gamma _{\tau _2}}dz' \int _{w' \in (1+\varepsilon )\gamma _{\tau _1}} dw' \\  &   \quad \frac{\exp \left( \frac{1}{r}\left( S(z;\tau _1,\chi _1)-S(w;\tau _2,\chi _2)+S(z';\tau _2,\chi _2)-S(w';\tau _1,\chi _1)\right) +O(1)\right) }{(z-w)(z'-w')} \end{aligned}$$

**Fig. 7:**
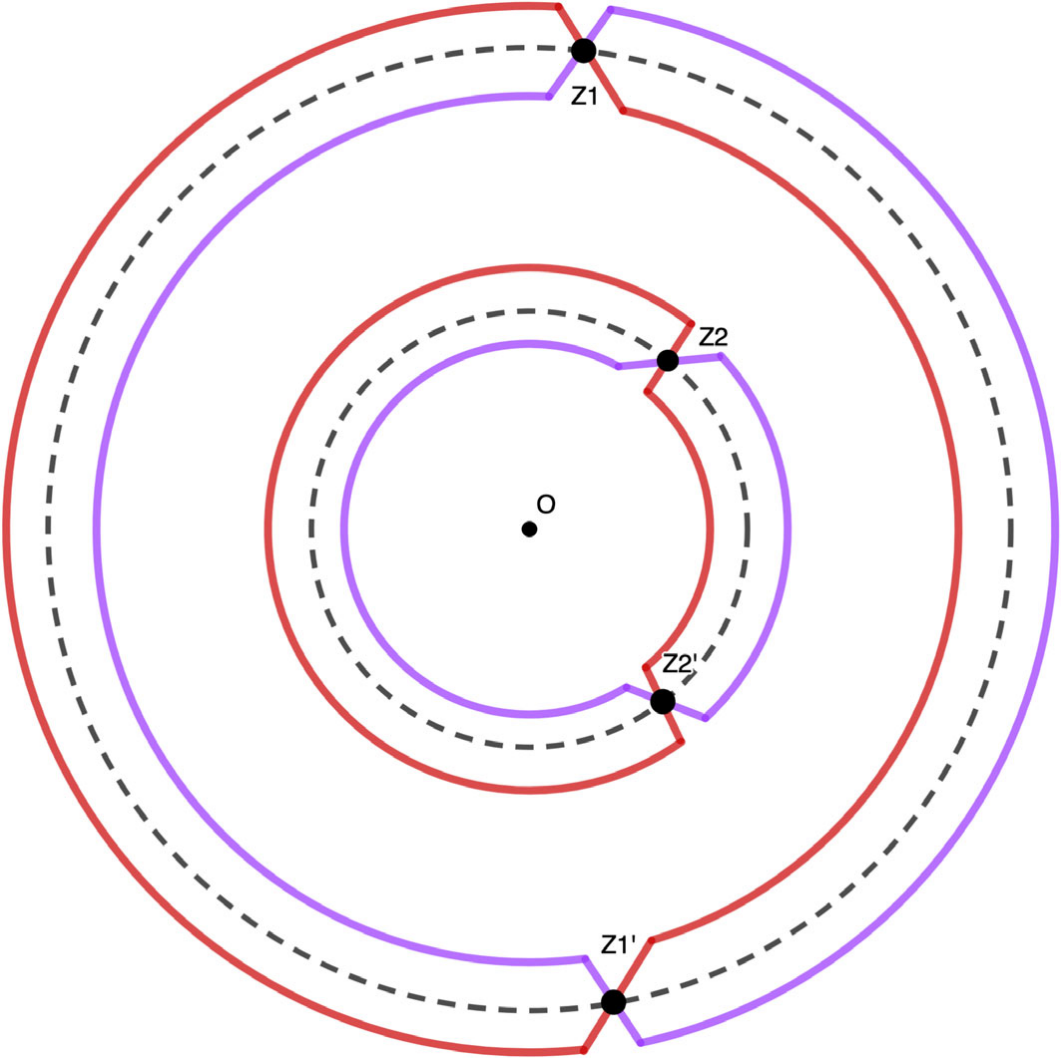
The contours $\gamma _{\tau _1}^>$, $\gamma _{\tau _1}^<$ are the thick contours near the dotted circle with the largest radius, the circle $\gamma _{\tau _1}$; the contours $\gamma _{\tau _2}^>$ and $\gamma _{\tau _2}^<$ are the thick contours near the dotted circle with the smallest radius, the circle $\gamma _{\tau _2}$

We first consider the case when $\tau _1 \ne \tau _2$, and by symmetry, we assume that $\tau _1 > \tau _2$. One can then deform the contours as previously. Precisely, we now integrate over

$$\begin{aligned} z \in \gamma _{\tau _1}^<, \hspace{0.1cm} w \in \gamma _{\tau _2}^>, \hspace{0.1cm} z' \in \gamma _{\tau _2}^<, \hspace{0.1cm} w' \in \gamma _{\tau _1}^> \end{aligned}$$

in order to have

$$\begin{aligned} \mathfrak {R}\left( S(z;\tau _1,\chi _1)-S(w';\tau _1,\chi _1) \right)<0, \quad \text {and} \quad \mathfrak {R}\left( S(z';\tau _2,\chi _2)-S(w;\tau _2,\chi _2) \right) <0, \end{aligned}$$

see Figure 7. These deformations do not affect the value of the integrals, because they involve separate variables. For all $\delta \in (0,1)$, we have

51 $$\begin{aligned} \frac{\exp \left( \frac{1}{r}\left( S(z;\tau _1,\chi _1)-S(w;\tau _2,\chi _2)+S(z';\tau _2,\chi _2)-S(w';\tau _1,\chi _1)\right) \right) }{\exp (-r^{-\delta })} \rightarrow 0 \end{aligned}$$

as $r \rightarrow 0$, for all $z,z',w,w'$ in the new contours except at a finite number of points. If the new contours $\gamma _{\tau _1}^<$ and $\gamma _{\tau _1}^>$ (resp. $\gamma _{\tau _2}^<$ and $\gamma _{\tau _2}^>$) differ from the circle $\gamma _{\tau _1}$ (resp. $\gamma _{\tau _2}$) by a distance less than $\varepsilon >0$, we have $|u-v|> |e^{\tau _1/2} -e^{\tau _2/2} | - 2\varepsilon$, for $u \in \gamma _{\tau _1}^< \cup \gamma _{\tau _1}^>$ and $v \in \gamma _{\tau _2}^< \cup \gamma _{\tau _2}^>$ from which we obtain the domination

$$\begin{aligned} \left| \frac{\exp \left( \frac{1}{r}\left( S(z;\tau _1,\chi _1)-S(w;\tau _2,\chi _2)+S(z';\tau _2,\chi _2)-S(w';\tau _1,\chi _1)\right) \right) }{(z-w)(z'-w')} \right| \le \frac{C}{|e^{\tau _1/2}-e^{\tau _2/2}|^2} \end{aligned}$$

for all $z,z',w,w'$ in the new contours except at a finite number of points, where *C* is large enough. Recalling (51), the proof for the case $\tau _1 >\tau _2$ is complete.

**Fig. 8:**
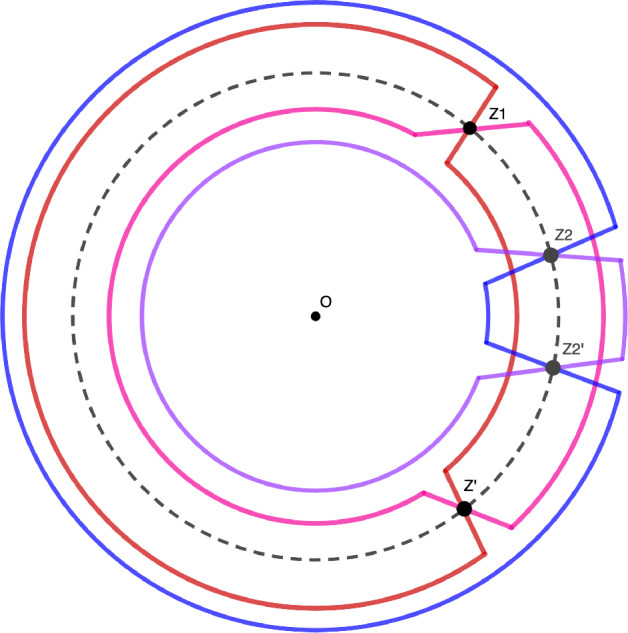
The thick contours $\gamma _{\tau }^{>,1}$ and $\gamma _\tau ^{<,1}$ cross the dotted circle $\gamma _\tau$ at points $z1=e^\tau z(\tau ,\chi _1)$ and $z1'=e^\tau \overline{z(\tau ,\chi _1)}$, while the thick contours $\gamma _{\tau }^{>,2}$ and $\gamma _{\tau }^{<,2}$ cross the circle $\gamma _\tau$ at points $z2=e^\tau z(\tau ,\chi _2)$ and $z2'=e^\tau \overline{z(\tau ,\chi _2)}$

For $\tau _1=\tau _2=\tau$, the proof is as follows. One deforms the contours as for the preceding case, but now, the deformations affect the value of the kernel since we can not avoid the residues at $z=w$ and $z'=w'$. We have, for example, the following case

52 $$\begin{aligned} \begin{aligned} \mathcal {K}&_{e^{-r}}\left( \frac{1}{r}(\tau ,\chi _1)+(t_1^1,h_1^1); \frac{1}{r}(\tau ,\chi _2)+(t_2^1,h_2^1)\right) \\&\mathcal {K}_{e^{-r}}\left( \frac{1}{r}(\tau ,\chi _2)+(t_1^2,h_1^2); \frac{1}{r}(\tau ,\chi _1)+(t_2^2,h_2^2)\right) \\\&=(1+O(1))\left( \frac{1}{(2i\pi )^2}\int _{z {\in } \gamma _\tau ^{<,1}}\int _{w {\in } \gamma _\tau ^{>,2}}... +\frac{1}{2i\pi }\int _w Res_{z=w} f(z,w;\tau ,\chi _1,\chi _2)dw \right) \\&\quad \times \left( \frac{1}{(2i\pi )^2} \int _{z' \in \gamma _\tau ^{<,2}}\int _{w' \in \gamma _\tau ^{>,1}}... + \frac{1}{2i\pi }\int _{w'} Res_{z'=w'}f(z',w';\tau ,\chi _2,\chi _1)dw' \right) , \end{aligned}\nonumber \\ \end{aligned}$$

where

53 $$\begin{aligned}  &   \int _w Res_{z=w}f(z,w;\tau ,\chi _1,\chi _2)dw\nonumber \\  &   \quad =\int _{|w|=e^{\tau /2}, \hspace{0.1cm} |\arg (w)|<\phi _{\tau ,\chi _2}} \frac{(q^{1/2+\tau /r+t_2}w;q)_\infty }{(q^{1/2+\tau /r+t_1}w;q)_\infty }\frac{dw}{w^{1/r(\chi _1-\chi _2)+h_1^1-h_2^1 + t_1^1-t_2^1}}, \end{aligned}$$

and

54 $$\begin{aligned}  &   \int _{w'} Res_{z'=w'}f(z',w';\tau ,\chi _2,\chi _1)dw\nonumber \\  &   \quad =\int _{|w'|=e^{\tau /2}, \hspace{0.1cm} |\arg (w')|<\phi _{\tau ,\chi _2}} \frac{(q^{1/2+\tau /r+t_2}w';q)_\infty }{(q^{1/2+\tau /r+t_1}w';q)_\infty }\frac{dw'}{w'^{1/r(\chi _2-\chi _1)+ h_1^2-h_2^2 + t_1^2-t_2^2}},\nonumber \\ \end{aligned}$$

the argument $\phi _{\tau ,\chi _2}$ being an argument of $z(\tau ,\chi _2)$ (see Figure 8). Equality (52) is valid when

$$\begin{aligned} t_1^1 \ge t_2^1, \hspace{0.1cm} t_1^2 \ge t_2^2, \hspace{0.1cm} \text {and } \arg \left( z(\tau ,\chi _1) \right) <\arg \left( z(\tau ,\chi _2) \right) , \end{aligned}$$

and the other cases can be treated in a similar way. Note that from (49), the factor

$$\begin{aligned} f_r(w):=\frac{(q^{1/2+\tau /r+t_2}w;q)_\infty }{(q^{1/2+\tau /r+t_1}w;q)_\infty } \end{aligned}$$

goes to

$$\begin{aligned} (1-e^{-\tau }w)^{t_1-t_2} \end{aligned}$$

as *r* tends to 0, uniformly in *w* on compact sets. Thus, the derivative $f_r'(w)$ converges as *r* tends to 0, and is in particular bounded independently of *r*. Integrating (53) and (54) by parts leads to

55 $$\begin{aligned}  &   \left| \int _w Res_{z=w}f(z,w;\tau ,\chi _1,\chi _2)dw \right| \nonumber \\  &   \quad \le C \frac{\exp \left( \frac{\tau }{2r}(\chi _2-\chi _1)\right) }{1/r|\chi _1-\chi _2|} +\frac{\exp \left( \frac{\tau }{2r}(\chi _2-\chi _1)\right) }{1/r|\chi _1-\chi _2|}\nonumber \\  &   \qquad \left| \int _{|w|=1, \hspace{0.1cm} \arg (w)<\phi _{\tau ,\chi _2}}\,f_r'(e^{-\tau /2}w)\frac{dw}{w^{1/r(\chi _1-\chi _2)+\Delta h + \Delta t-1}}\right| \nonumber \\  &   \quad \le C\frac{r}{|\chi _1-\chi _2|}\exp \left( \frac{\tau }{2r}(\chi _2-\chi _1)\right) , \end{aligned}$$

and

56 $$\begin{aligned} \left| \int _{w'} Res_{z'=w'}f(z',w';\tau ,\chi _2,\chi _1)dw \right| \le C\frac{r}{|\chi _1-\chi _2|}\exp \left( \frac{\tau }{2r}(\chi _1-\chi _2)\right) . \end{aligned}$$

It is clear that, by construction, we have

57 $$\begin{aligned} \left| \int _{z \in \gamma _\tau ^{<,1}}\int _{w \in \gamma _\tau ^{>,2}}... \right| \le C \exp \left( \frac{\tau }{2r}(\chi _2-\chi _1)\right) , \end{aligned}$$

and

58 $$\begin{aligned} \left| \int _{z' \in \gamma _\tau ^{<,2}}\int _{w' \in \gamma _\tau ^{>,1}}... \right| \le C \exp \left( \frac{\tau }{2r}(\chi _1-\chi _2)\right) . \end{aligned}$$

We now expand the product in (52). The term

$$\begin{aligned} \int _{z \in \gamma _\tau ^{<,1}}\int _{w \in \gamma _\tau ^{>,2}}... \times \int _{z' \in \gamma _\tau ^{<,2}}\int _{w' \in \gamma _\tau ^{>,1}}... \end{aligned}$$

is by construction dominated by any polynomial in *r*. The estimates (55) and (56) imply that the product of the integrals of the residues is smaller than

$$\begin{aligned} \frac{Cr^2}{|\chi _1-\chi _2|^2}, \end{aligned}$$

while the combinations of (55) and (58), and (56) and (57) entail that the remaining terms are smaller than

$$\begin{aligned} \frac{Cr}{|\chi _1-\chi _2|}. \end{aligned}$$

Lemma 4.1 is proved.

#### Proof of Lemma 4.2

Lemma 4.2 is proved using Propositions 1.6 and 4.3. Let $\mathcal {K} \subset \mathbb {R}^2$ be a compact set, and let $m,m' \subset E$ be finite. Let $(\tau ,\chi ) \in A\cap \mathcal {K}$. By Proposition 1.6, we have

$$\begin{aligned}  &   \mathbb {E}_r \left[ c_{\frac{1}{r}(\tau ,\chi )+m} c_{\frac{1}{r}(\tau ,\chi )+m'}\right] - \mathbb {E}_r \left[ c_{\frac{1}{r}(\tau ,\chi )+m}\right] \mathbb {E}_r \left[ c_{\frac{1}{r}(\tau ,\chi )+m'} \right] \\  &   \quad = \mathbb {E}_{(\tau ,\chi )} \left[ c_{m} c_{m'}\right] - \mathbb {E}_{(\tau ,\chi )} \left[ c_m\right] \mathbb {E}_{(\tau ,\chi )} \left[ c_{m'} \right] +O(r). \end{aligned}$$

Now, by Proposition 4.3, the function

$$\begin{aligned} (\tau ,\chi ) \mapsto \mathbb {E}_{(\tau ,\chi )} \left[ c_{m} c_{m'}\right] - \mathbb {E}_{(\tau ,\chi )} \left[ c_m\right] \mathbb {E}_{(\tau ,\chi )} \left[ c_{m'} \right] \end{aligned}$$

is bounded, as long as $(\tau , \chi )$ belong to a compact set. Lemma 4.2 is proved, and Theorem 1.8 is completely proved.

## Data Availability

Data sharing is not applicable to this article as no datasets were generated or analyzed during the current study.
